# The *C. elegans* eggshell

**DOI:** 10.1895/wormbook.1.179.1

**Published:** 2018-08-02

**Authors:** Kathryn K. Stein, Andy Golden

**Affiliations:** 1 Laboratory of Biochemistry and Genetics, National Institute of Diabetes, Digestive, and Kidney Diseases, National Institutes of Health, Bethesda, Maryland 20892, USA

## Abstract

In all animals, oocytes are surrounded by an extracellular matrix upon fertilization. This matrix serves similar purposes in each animal. It functions to mediate sperm binding, to prevent polyspermy, to control the chemical environment of the embryo, and to provide physical protection to the embryo as it developes. The synthesis of the *C. elegans* matrix, or eggshell, begins when the oocyte enters the spermatheca and is fertilized by a single sperm. The process of eggshell synthesis is thought to take place during the completion of the maternal meiotic divisions such that the multi-layered eggshell is completed by anaphase II. The synthesis of the eggshell occurs in a hierarchical pattern such that the outermost layers are synthesized first in order to capture and retain the innermost layers as they form. Recent studies have revealed that the lipid-rich permeability barrier is distinct from the outer trilaminar eggshell. These new findings alter our previous understanding of the eggshell. This chapter aims to define each of the eggshell layers and the molecules that are known to play significant roles in their formation.

In all vertebrate and invertebrate species, oocytes are surrounded by an extracellular matrix (ECM) that is extensively modified upon fertilization. In each organism, the ECM serves similar purposes: (1) to mediate sperm binding, (2) to prevent the entry of supernumerary sperm, (3) to control the chemical environment of the embryo, and (4) to provide physical protection to the embryo as it encounters the environment in which it will develop. Although the function of the embryonic ECM is retained across species, it is not known whether the composition is conserved since the proteins that make up these structures are largely undefined. Generally, however, the ECM of many species is composed of glycoproteins. Also common to many organisms is a regulated exocytic event that is triggered after fertilization to build or modify the ECM (Wessel et al., 2001). In this chapter, we will describe the current understanding of the composition and synthesis of the *C. elegans* eggshell, highlighting the function of each layer and the genes known to be required for their assembly.

## 1. Overview of the structure of the *C. elegans* eggshell

In the sections below, we will suggest some nomenclature changes based on conversations with those in the field and reviewers of this chapter. The first being that we number each layer according to the order in which they are deposited into the eggshell. The second suggestion is that we rename the spaces as layers as well.

**Figure 1:**
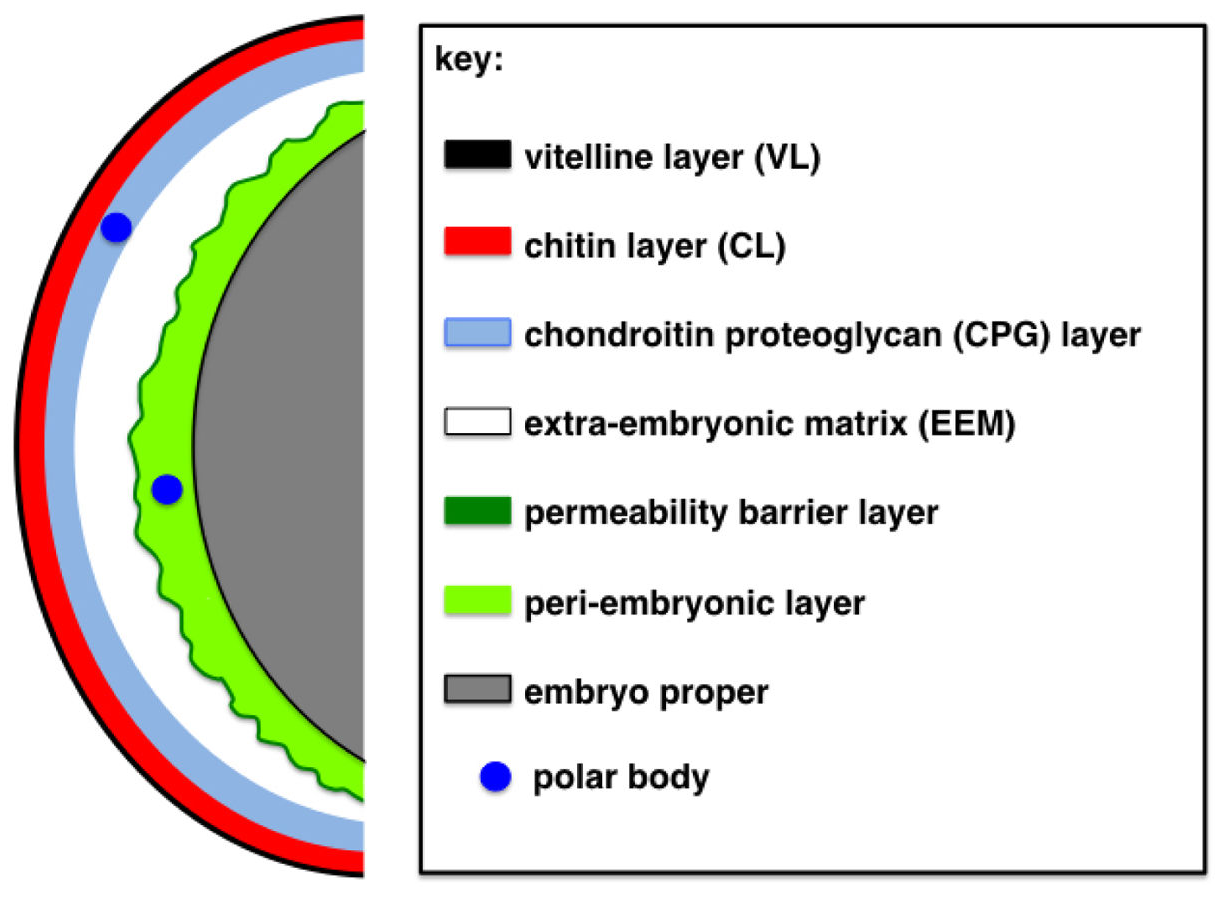
Schematic of the layers of the *C. elegans* eggshell. Shown is the trilaminar eggshell consisting of an outermost vitelline layer (VL; black), a middle chitin layer (CL; red), and an innermost chondroitin proteoglycan layer (CPG; blue). Separating the trilaminar outer eggshell from the inner permeability barrier (dark green) is the extra-embryonic matrix (EEM; white). Under the permeability barrier layer is an amorphous peri-embryonic layer (light green), which coats the embryo proper (gray). The plasma membrane of the embryo is shown as a black line. The peri-embryonic layer is most obvious at the anterior of the embryo. The polar bodies are shown in dark blue; the first polar body is immobilized in the CPG layer of the trilaminar outer eggshell, while the second polar body is mobile in the peri-embryonic layer. The position of the polar bodies is indicative of both the times at which the meiotic divisions are occurring and at which each eggshell layer is being synthesized.

For many years, the *C. elegans* eggshell was known to be composed of three strata (Hall and Altun, 2008; Rappleye et al., 1999; Wharton, 1980; Bird, 1971) and the perivitelline space [now referred to as the extra-embryonic matrix (EEM), as suggested by a recent review (Johnston and Dennis, 2012)]. Two additional layers have recently been observed and defined (Olson et al., 2012; Benenati et al., 2009)(Figure 1). These layers are defined visually through electron micrographs and also by diagnostic biochemical treatments that can remove individual layers from the embryo (Olson et al., 2012; Benenati et al., 2009; Rappleye et al., 1999; Schierenberg and Junkersdorf, 1992). The outermost layer is the vitelline layer (VL) and can be selectively removed by treatment with sodium hypochlorite, leaving the remainder of the eggshell intact. This layer is present on the oocyte surface prior to fertilization. The next layer is the chitin layer (CL), digestible by chitinase. When this layer is removed, the embryo loses its ovoid shape and rounds up into a sphere. The third layer was previously thought to be a lipid-rich layer, based on chemical composition experiments done in *Ascaris* (Foor, 1967). However, Olson et al. (2012) have recently shown that this third layer is composed of proteoglycans. It is now referred to as the chondroitin proteoglycan (CPG) layer (Olson et al., 2012). The three outer layers are now known as the trilaminar outer eggshell. The fourth layer was previously known as the perivitelline space because it originally sat adjacent to the vitelline membrane, a name no longer used for the fifth layer (see Section 6 and Section 7). Johnston and Dennis (2012) suggested calling this layer the extra-embryonic matrix (EEM), a name we will use throughout this chapter. The EEM is an asymmetric layer that sits below the CPG layer (of the trilaminar outer eggshell). This layer is larger at the anterior end where the first extruded polar body resides. It is thought that this layer is fluid-filled and contains a number of proteins (Olson et al., 2012; Johnston et al., 2006). The fifth layer, which is separated from the trilaminar outer eggshell by the EEM, is now thought to be the lipid-rich layer, which makes up the osmotic/permeability barrier for the developing embryo. Olson et al. (2012) refer to this layer as the permeability barrier and its function is to keep out most small molecules and keep water in so the embryo stays hydrated. This layer has also been called the embryonic layer, due to its close apposition to the embryo (Benenati et al., 2009). Residing between the permeability barrier layer and the embryo proper is an amorphous space now called the peri-embryonic layer (layer six; Figure 1) (Olson et al., 2012). While Benenati et al. refer to the entire region as the embryonic layer, Olson *et* al. distinguish the permeability barrier as a thin membrane-like structure that encloses the peri-embryonic layer beneath it. Please refer to Section 7 for an expanded definition of these two layers. All of these layers are thought to be synthesized by the embryo in a hierarchical assembly pattern (Olson et al., 2012). Another layer has been observed in numerous nematode eggshells and has been called the uterine layer, the uterine coat, or the post-ovulatory envelope (Wharton, 1980; Foor, 1967). This layer is secreted by uterine cells as the embryos pass through the uterus. All of these layers will be discussed in greater detail in Sections 3 through 7, and 9.

## 2. Timeline for eggshell formation

The impermeable eggshell is established by the end of anaphase II of meiosis, with final modifications being made as the embryo undergoes its first few mitotic divisions (Bembenek et al., 2007; Zhang et al., 2005). From studies with *Ascaris*, the vitelline layer is present on the oocytes in the oviduct (Foor, 1967). Upon entry into the spermatheca, the oocyte, which is in prophase of meiosis I, is fertilized by a single sperm. A single calcium wave is the earliest event observed in the spermatheca (Samuel et al., 2001); this calcium spike lasts 2–4 seconds. Recently, Jun Takayama and Shuichi Onami probed the fertilization-induced Ca ^2+^ transient in *C. elegans* oocytes and found that fertilization triggers biphasic Ca ^2+^ waves in oocytes. In addition to a fast local wave that appeared at the site of sperm entry, similar to that observed by Samuel et al. (2001), a slow global wave was also observed to spread from this site to the opposite pole (Jun Takayama and Shuichi Onami, personal communication).

Each ovulated oocyte spends about 3–5 minutes in the spermatheca. Some time during this period it is fertilized before passing through the spermathecal/uterine valve into the uterus. In the uterus, the maternal chromosomes undergo both meiotic divisions in the span of 20 minutes; the first mitotic division takes place 40 minutes after fertilization (at 20 C). Evidence of the chitin layer of the eggshell has been observed in the spermatheca (Maruyama et al., 2007). Fertilization is the most likely trigger for the activation of the enzyme chitin synthase (CHS-1), responsible for the synthesis of the chitin layer. Synthesis of chitin is not dependent on cell cycle progression: zygotes arrested in metaphase of the first meiotic division still show normal chitin distribution on the +1 zygote (+1 refers to the position of the newly fertilized zygote relative to the spermatheca; the −1 is the oocyte adjacent to the spermatheca that is the next one to be fertilized) (Johnston et al., 2010). The formation of the third layer, the chondroitin proteoglycan layer, does depend on cell cycle progression. Cortical granule exocytosis (CGE), which takes place during anaphase I, is responsible for establishing this layer (Olson et al., 2012; Bembenek et al., 2007). The next layer, which is separated from the outer three layers by the EEM, is the lipid-rich permeability barrier. It is synthesized during meiosis II (Olson et al., 2012). The zygote thus becomes impermeable to dyes by anaphase II (see Figure 1 for a schematic of these layers).

## 3. Layer one: the vitelline layer

Very little is known about the origin or the composition of the vitelline layer (VL). Much of what we know about this layer comes from studies with *Ascaris lumbricoides*. There is some agreement that this layer is present on the oocyte and that it is maintained post-fertilization (Wharton, 1980; Foor, 1967). It appears to be coincident with the plasma membrane in the oocyte, but separates from it upon fertilization (Hall and Altun, 2008; Foor, 1967). This layer might mediate sperm-oocyte binding, similar to what has been observed in other species. How the sperm successfully penetrates this layer in *C. elegans* is unknown. There are no published observations of sperm-egg fusion or the subsequent fusion site to shed light on how this penetration might occur. Although there is a report of some lectin staining enriched in the VL (Bembenek et al., 2007), there is not, to our knowledge, a way to distinguish it from the plasma membrane of the oocyte. This layer can be removed from mitotic embryos using sodium hypochlorite (Schierenberg and Junkersdorf, 1992) without harming their development.

The *cbd-1* (chitin-binding domain protein; H02I12.1) gene encodes the only known VL marker to date. This gene is essential for embryonic development (Johnston et al., 2010). CBD-1::GFP is localized to the surface of developing oocytes and embryos (Johnston et al., 2010). Observations from our lab with a transgenic CBD-1::mCherry line support the conclusions that CBD-1 is a vitelline layer marker. CBD-1::mCherry can be stripped off the embryo with hypochlorite treatment, further supporting the conclusion that it is a component of the vitelline layer (D. N. Levine, K. K. Stein, and A. Golden, unpublished observations).

CBD-1-depleted oocytes exhibited precocious phospho-histoneH3 antibody staining and the ovulated oocytes are small, often pinched in two as they enter the uterus, indicative of possible eggshell defects (Johnston et al., 2010). CBD-1 also appears to have a role in the proper formation of the eggshell as embryos from *cbd-1* RNAi-treated hermaphrodites are fertilized by more than one sperm, and produce an eggshell that is misshapen with chitin localized only at one end of the embryo. Furthermore, CBD-1 is required for membrane localization of EGG-1 and EGG-2 on the oocyte (Johnston et al., 2010), which are, in turn, essential for the membrane localization of CHS-1, EGG-3 and MBK-2 (see Section 4).

## 4. Layer two: the chitin layer

**Figure 2:**
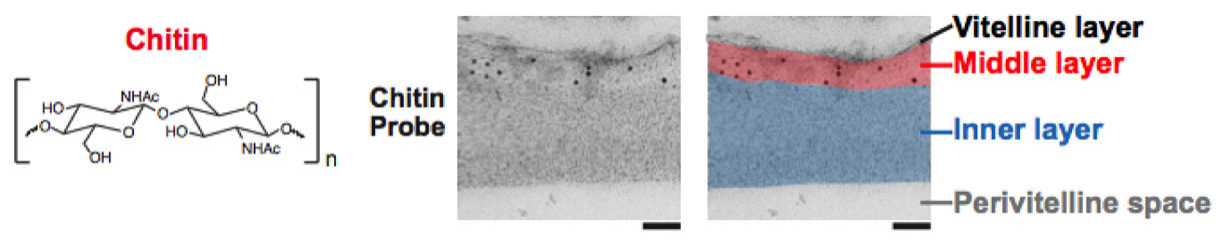
Chitin is the middle layer of the outer trilaminar eggshell. Chitin is composed of repeating units of β-(1,4)-linked *N*-acetylglucosamine. Image is an electron micrograph of an eggshell after immunogold labeling (10-nm gold beads) with a chitin-binding probe. Bars, 100 nm. Figure and legend are from Olson et al., 2012. Reprinted with permission from The Journal of Cell Biology. The perivitelline space will be called the extra-embryonic matrix throughout this chapter.

Chitin is a polymer of β-(1,4)-linked N-acetyl-glucosamine residues (Figure 2) that is prevalent in the support structures of fungi (cell wall), nematode embryos (eggshell and pharynx), and arthropods (exoskeleton) (Merzendorfer, 2006). In nematodes, chitin has long been implicated as a component of the eggshell because treatment of the embryo with chitinase is necessary for the removal of the eggshell (Edgar, 1995; Wharton, 1980). The chitin layer (CL) provides the mechanical support for the oval embryo; embryos without chitin are round and misshapen. The chitin eggshell can be thought of as a meshwork or sponge-like structure. It is a porous, but rigid, structure that gives the embryo its ovoid shape.

The presence of chitin in the nematode eggshell has been directly demonstrated using either a fluorescently-conjugated or immunogold-labeled chitin-binding domain (CBD) probe (New England Biolabs) in *C. elegans* (Olson et al., 2012; Parry et al., 2009; Maruyama et al., 2007; Johnston et al., 2006) (Figure 1 and Figure 2). Chitin is evident in the eggshell of a newly fertilized zygote at metaphase I (Maruyama et al., 2007). Chitin is not detectable on oocytes, and is dynamically modified in the early embryo: the fluorescent CBD probe can easily detect chitin in the eggshell of the +1 zygote, but this binding diminishes with subsequent embryonic development, beginning at the 2-cell stage (Zhang et al., 2005). Treatment of embryos with sodium hypochlorite restores CBD probe binding throughout embryogenesis, indicating that the chitin layer is modified by the outer vitelline layer or a sodium hypochlorite-sensitive substance that is present on the zygote after its exit from the spermatheca (Zhang et al., 2005). Interestingly, WGA labeling of the eggshell brightens as the embryo ages, and thus serves as another marker for eggshell modification (Bembenek et al., 2007). The most likely source of modifications are proteins that are being secreted by the uterine cells and binding to the eggshell. To ask whether the chitin layer modifications of the eggshell are affected by vesicle trafficking, Sato et al. (2008) stained control, metaphase I-arrested zygotes, and RAB-11.1-depleted embryos with the CBD probe. Rab GTPases play key roles in vesicle transport in many organisms: of the 30 *rab* genes in *C. elegans*, RAB-11.1 depletion strongly inhibited cortical granule exocytosis (but not cortical granule formation). In control and metaphase I-arrested zygotes, the CBD probe is present on the +1 zygote and then disappears by the +2 embryo stage. This observation suggests that in the absence of cortical granule fusion (in the metaphase I-arrested zygotes), the modification of the chitin layer still occurs normally. Thus, whatever is responsible for this chitin modification is still present when cortical granule exocytosis is blocked. However, RAB-11.1-depleted embryos had heavy and persistent labeling of the eggshell with the CBD probe in all embryos. These findings suggest that the remodeling of the CL also relies on a RAB-11.1-dependent vesicle transport pathway that does not include the cortical granules.

The genes required for the synthesis of the CL all share a common phenotype when depleted. Embryos lacking the CL of the eggshell are misshapen and extremely fragile. The fertilized zygotes are often crushed as they exit the spermatheca; this is in stark contrast to an unfertilized oocyte, which can pass into the uterus with little damage. Chitin-deficient zygotes fail to extrude polar bodies even though they undergo the meiotic divisions. Most embryos accumulate in the uterus as an indistinct massthose that make it out of the spermatheca and uterus without being crushed are multinucleate, polyploid one-cell embryos that fail to undergo cytokinesis. It should be no surprise that such embryos also do not establish embryonic polarity (Benenati et al., 2009; Johnston et al., 2006). The polarity phenotypes described for many eggshell synthesis genes could be due to the observation that polarity cues depend on microtubules. Embryos that fail polar body extrusion are left with an excess of chromosomes and microtubules. Such embryos would be expected to display defects in polarity. Even one-cell arrested embryos have polarity defects because of the persistence of the meiotic spindle (Wallenfang and Seydoux, 2000). Thus, it is likely that the meiotic phenotypes and polarity defects in many eggshell mutants are indirect, caused by the lack of a chitinous eggshell, rather than a result of a specific role of chitin synthesis genes in these processes.

Below is a description of a number of proteins involved in the synthesis of the CL of the eggshell.

### 4.1. CHS-1

*C. elegans* has two genes, *chs-1* and *chs-2*, that encode chitin synthase, the enzyme that polymerizes UDP-N-acetyl-glucosamine into chitin (Zhang et al., 2005; Veronico et al., 2001). Animals homozygous for the deletion mutation, *chs-1(ok1120)* or depleted of CHS-1 by RNAi do not produce any viable progeny. In contrast, depletion of the somatically-expressed CHS-2 results in pharyngeal defects in larvae (Zhang et al., 2005). These data suggest that *chs-1* is likely the gene responsible for chitin synthesis in the embryonic eggshell. In the mutant *chs-1(ok1120)*, no embryonic chitin was detected with the CBD probe*,* consistent with CHS-1 being the sole synthase in the embryo (Zhang et al., 2005). Embryos produced by *chs-1* hermaphrodites are round rather than oval, do not divide, and are permeable to dye, all characteristics of an Osmotic Integrity Defective (OID) phenotype. These chitin-free eggshells are dye-permeable because the chitin layer is essential to catch the exocytosed contents of the innermost layers of the eggshell; the chitin layer itself does not serve as a permeability barrier (see Section 7).

The main player in chitin synthesis in the *C. elegans* eggshell is the enzyme chitin synthase, CHS-1. CHS-1 resides in membranes and is predicted to have 15 transmembrane domains (Veronico et al., 2001). Available data and comparisons with other glycosyltransferases, such as hyaluronan synthase, suggest that that these proteins may form multimers in the plasma membrane, creating a channel through which the nascent chitin is translocated across the membrane (Merzendorfer, 2006). There are two known mechanisms of chitin assembly. In yeast, there are vesicular structures called chitosomes, which synthesize and store chitin until the vesicles are triggered to fuse with the plasma membrane (see Bartnicki-Garcia, 2006 for review). This is not likely to be the route of synthesis in the *C. elegans* embryo. The smooth cortical localization pattern of the GFP::CHS-1 fusion protein on the oocyte is consistent with plasma membrane residence (Maruyama et al., 2007) although there is no direct evidence at the ultrastructural level for the precise subcellular localization. In addition, there is currently no evidence for an early vesicle fusion event before anaphase I in *C. elegans*. Therefore, it is most likely that chitin is synthesized at the plasma membrane and translocated directly into the extracellular space where assembly into higher-order structures occurs. The amount of time between sperm fusion and the formation of the eggshell is very rapid, about five minutes (Maruyama et al., 2007; Ward and Carrel, 1979). Comparable events occur after cell division in yeast to generate the new cell wall: Chs2p, the CHS-1 yeast ortholog, is at the budneck for only 8 minutes in yeast (Zhang et al., 2006). By anaphase of meiosis I in the +1 *C. elegans* zygote, CHS-1 moves to cytoplasmic foci (Maruyama et al., 2007). The sequential synthesis of the eggshell layers become apparent at this time; a switch between the assembly of the CL and Layer Three, the CPG layer, occurs when CHS-1 is internalized from the cell surface (Olson et al., 2012). This internalization occurs at the time that the cortical granules exocytose their contents at anaphase of meiosis I (see Section 5) (Olson et al., 2012).

### 4.2. GNA-2

Chitin synthase is the last enzyme in the biosynthetic pathway of chitin. The enzymatic pathway that generates UDP-GlcNAc, the substrate for chitin synthase, is the hexosamine pathway. This pathway consists of at least five enzymes that are essential for the synthesis of chitin from glucose-6-phosphate, and individual depletion of four of these enzymes by RNAi has been reported to generate an OID phenotype (Johnston and Dennis, 2012; Johnston et al., 2006; Sonnichsen et al., 2005). The best-studied of these genes is *gna-2*, which codes for glucosamine 6-phosphate N-acetyltransferase. Hermaphrodites homozygous for the deletion allele *gna-2(qa705)* are 100% Maternal Effect Lethal (Mel) and lay round, osmotically-sensitive one-cell embryos (Johnston et al., 2006). In addition, the sperm pronucleus centrosome complex (SPCC) never associates with the cortex and pseudocleavage furrows are not observed (Johnston et al., 2006). When a transgene is used to rescue this deletion, viability is partially restored, but a High Incidence of Males (Him) phenotype is observed, suggesting defects in the segregation of chromosomes during the meiotic divisions. However, it is not clear how a Him phenotype would be directly linked to eggshell synthesis. GNA-2 is also required for the glycosylation of proteins that enter the secretory pathway, so a cohort of uncharacterized glycosylated proteins may be essential for normal chromosome segregation.

### 4.3. EGG-1, EGG-2

Mutations that result in broods of unfertilized oocytes are fertilization-defective due to either defects in the oocyte or the sperm. Mutations or RNAi depletion of the *egg* genes demonstrate a function for these genes in fertilization of the oocyte (Kadandale et al., 2005; Lee and Schedl, 2001; Maeda et al., 2001). EGG-1 and EGG-2 are LDL receptor repeat-containing proteins (Kadandale et al., 2005). GFP fusions of either protein are localized to the oocyte plasma membrane and are removed from the surface by endocytosis after fertilization (Johnston et al., 2010). When CHS-1, GNA-2, or EGG-3 (see Section 4.4) are depleted, EGG-1::GFP remains on the plasma membrane even after fertilization and passage through the spermatheca. Thus the construction of the chitin layer of the eggshell must promote the endocytosis and removal of EGG-1 from the oocyte surface (Johnston et al., 2010). RNAi experiments suggest that EGG-1 and EGG-2 function redundantly (Kadandale et al., 2005). The roles of EGG-1 and EGG-2 remain conflicted. One report states that animals depleted of both EGG-1/2 produce oocytes that are fertilization-incompetent despite passage through the spermatheca (Kadandale et al., 2005). Earlier RNAi studies with just *egg-1* at 25 C came to the same conclusion (Lee and Schedl, 2001; Maeda et al., 2001). The most recent report states that animals depleted of both EGG-1/2 produce oocytes that are fertilized, but are defective in the formation of the chitin layer of the eggshell and the block to polyspermy (Johnston et al., 2010). There is no satisfactory explanation for these conflicted observations and thus the exact role of these proteins remains unresolved.

### 4.4. EGG-3, EGG-4, EGG-5

Three additional *egg* genes function post-fertilization. EGG-3, EGG-4 and EGG-5 are protein tyrosine phosphatase-like proteins (PTPL) that likely do not act as phosphatases: they contain a phosphatase domain, but are missing key catalytic residues required for phosphatase activity. This class of proteins is termed pseudo-phosphatases. Hermaphrodites depleted of EGG-3 by deletion mutation or RNAi produce no viable progeny (Maruyama et al., 2007) although fertilization and the block to polyspermy appear to occur normally. This contrasts with a recent report that 29% of EGG-3-depleted embryos are polyspermic when in a compromised genetic background (Johnston et al., 2010). The role of EGG-3 in the block to polyspermy remains controversial and should be resolved by further examination. Importantly, chitin eggshell formation is dependent on EGG-3 (Johnston et al., 2010; Maruyama et al., 2007). Meiosis I proceeds normally without EGG-3, but no polar bodies are extruded and twelve univalents are frequently observed during meiosis II.

The localization pattern of EGG-3 is consistent with its role in eggshell formation. EGG-3 localizes to the oocyte plasma membrane, re-localizes to cytoplasmic foci at anaphase I, and is degraded subsequently. This redistribution was fertilization-independent, but cell cycle dependentre-localization did not occur in metaphase I-arrested zygotes (Maruyama et al., 2007). This observation reinforces the notion that a vast surface remodeling occurs with progression through the cell cycle. GFP::CHS-1 co-localizes with mCherry::EGG-3. Their normal localization patterns are interdependent: depletion of either disrupts the localization of the other. MBK-2, a kinase essential for the meiotic divisions and the oocyte-to-embryo transition (Robertson and Lin, 2013), also requires EGG-3 and CHS-1 for localization to the surface of the oocyte, but the reverse is not true (Johnston et al., 2010).

EGG-4 and EGG-5 are two redundant members of the PTPL class of proteins (with 99% amino acid identity). Similar to EGG-3, hermaphrodites treated with RNAi to deplete both proteins leads to penetrant embryonic lethality: embryos lack a rigid chitinous eggshell, they have defective actin caps (see Section 8), and polar body formation is defective (Parry et al., 2009). Polyspermy is observed in 16–25% of EGG-4/5 co-depleted or double mutant embryos (Parry et al., 2009). The EGG-4 and EGG-5 proteins localize to the oocyte plasma membrane with EGG-3 [and also in the nucleus (Cheng et al., 2009)]. During the transition from meiosis I to II, the EGG-4 and EGG-5 signal is lost from the surface and re-localizes to cortical speckles and around the male pronucleus (Cheng et al., 2009). EGG-3 and CHS-1 are required for the localization of EGG-4/5. EGG-4/5 in turn are required for cortical localization of MBK-2. Thus, the essential role of these pseudo-phosphatases, EGG-4 and EGG-5, is to sequester MBK-2 on the cortex until the meiotic divisions are initiated (Cheng et al., 2009). But why are EGG-4/5-depleted embryos polyspermic? The answer may be in the observation that CHS-1 and EGG-3 fail to come off of the cortex after fertilization (Parry et al., 2009). Since both are normally found in cytosolic foci at anaphase I, perhaps their persistence on the cortex of fertilized embryos is an indication that eggshell synthesis is compromised (as evident by the lack of chitin staining). Perhaps EGG-4/5 are responsible for the activation of CHS-1 and the polyspermy is simply due to the lack of a chitin layer. The complex interdependencies of all the EGG proteins, CHS-1, and MBK-2 for localization, and presumably enzymatic activity, highlight the essential roles of the CL in the block to polyspermy, and the proper resumption of meiosis and embryonic development.

### 4.5. SPE-11

SPE-11 is a protein expressed exclusively in sperm that is essential for the synthesis of the chitin layer of the eggshell after fertilization (Johnston et al., 2010). *spe-11* is the only strict paternal-effect lethal mutant in *C. elegans* to date *spe-11* males mated with wild-type hermaphrodites or females sire only dead embryos (Browning and Strome, 1996; Hill et al., 1989). The terminal SPE-11 phenotype is a multinucleate one-cell embryo without a functional eggshell. In these mutants, the chitin layer is observed as a small cap on the embryonic surface most closely apposed to the male pronucleus (Johnston et al., 2010; our unpublished observations). This cap may mark the site of sperm entry. The partial construction of the eggshell implicates SPE-11 in the transmission of the signal to form the eggshell upon fertilization. The machinery must include CHS-1, although its localization in *spe-11* mutants is unknown. EGG-3 does localize properly in a *spe-11* mutant (our unpublished observations). Johnson et al. (2010) have reported polyspermy in *spe-11(hc90)* embryos, indicative of a role for the eggshell in the block to polyspermy.

SPE-11 is expressed early in spermatogenesis where it is associated with chromosomes, and ultimately has a perinuclear localization pattern in mature sperm (Browning and Strome, 1996). SPE-11 has not been observed in fertilized embryos possibly due to dilution by the egg cytoplasm or rapid degradation (Browning and Strome, 1996; our unpublished observations). In sperm, SPE-11 does not appear to have a gross structural function: EM of *spe-11* mutant sperm revealed no obvious structural defects (Hill et al., 1989). Mutants produce sperm that are morphologically normal, motile, and competent to fertilize oocytes.

If SPE-11 has no obvious role in spermatogenesis, could it function if supplied exclusively through the oocyte? Browning and Strome (1996) had observed that maternal expression of SPE-11 (from a heat shock promoter) could rescue the *spe-11* lethality. Transgenic animals expressing *spe-11* under the germline-specific *pie-1* promoter exhibit expression in both sperm and oocytes (C. Merritt and G. Seydoux, personal communication). When *spe-11* mutant males are mated into this transgenic line, cross-progeny are observed and are viable (our unpublished observations). Thus, the successful construction of an eggshell when SPE-11 is provided by the oocyte demonstrates that localization of SPE-11 in the fertilizing sperm is not essential for its function. It is important to note that sperm-mediated delivery likely is an important regulatory mechanism to ensure that this essential protein does not promote eggshell formation until after fertilization. However, the observation that the *pie-1::spe-11* line is viable by itself suggests that SPE-11 expression in oocytes does not trigger precocious eggshell synthesis.

The phenotype of *spe-11* mutants is remarkably similar to that of *chs-1* and *gna-2* mutants, and supports the idea that SPE-11 may be the sperm signal to initiate or propagate eggshell synthesis upon fertilization. The genetic analysis to determine if any of the genes described above act downstream of *spe-11* has yet to be reported. The localization of these gene products in *spe-11* mutant embryos would also be very informative.

### 4.6. Other chitin-binding proteins

Johnston et al. (2006) reported eleven *C. elegans* genes that contain chitin-binding domains, one of which is described in Section 3 (CBD-1). Several of these have been implicated to play a role in eggshell synthesis based on RNAi depletion studies (Table 1, Section 12). Whether these chitin-binding proteins are expressed in the VL, the CL, or the CPG layer remains to be determined. Following the initial formation of the chitin layer of the eggshell, these chitin-binding proteins may serve to modify the properties of the eggshell, perhaps strengthening it or making it more stable. The observation that the detection of chitin (with a CBD probe) declines over developmental time could be due to the occupation of these sites by other chitin-binding proteins (Zhang et al., 2005).

## 5. Layer three: the CPG layer

**Figure 3:**
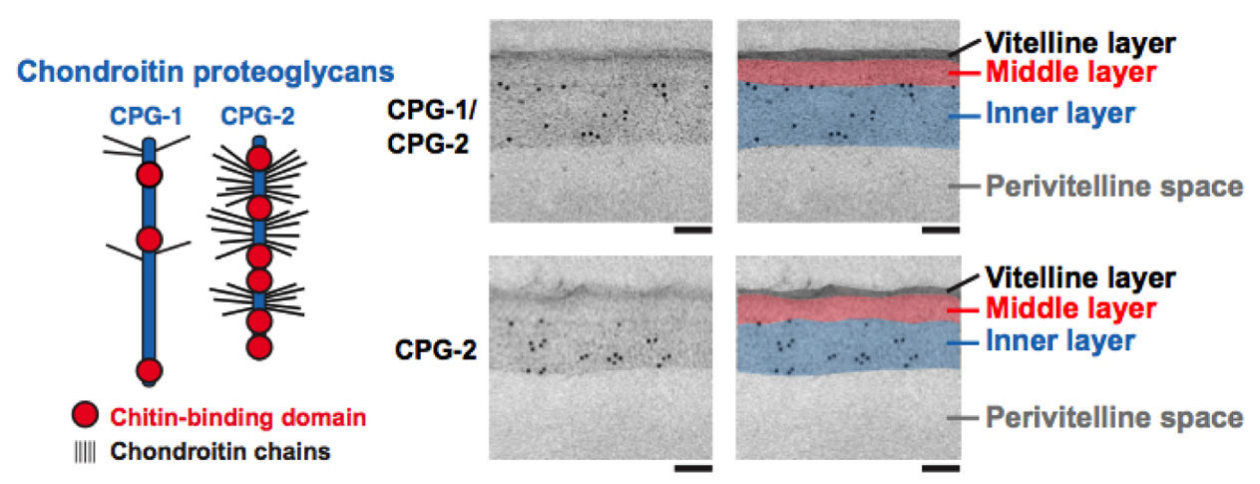
CPG-1 and CPG-2 are in the innermost layer of the trilaminar eggshell. CPG-1 and CPG-2 are secreted CPGs composed of a protein core (blue) with covalently linked chondroitin side chains (thin black lines are putative chondroitin attachment sites based on sequence consensus (Olson et al., 2006)) and chitin-binding domains. Images are electron micrographs of eggshells after immunogold labeling (10 nm beads) using antibodies recognizing both CPG-1 and CPG-2 (top) or specifically CPG-2 (bottom). Bars, 100 nm. Figure and legend are from Olson et al., 2012. Reprinted with permission from The Journal of Cell Biology. The perivitelline space will be called the extra-embryonic matrix throughout this chapter.

The third layer of the trilaminar outer eggshell had been thought to be a lipid-rich layer for the past 100 years or more. The reason this layer was thought to be lipid-rich was based on chemical extraction and analysis (Chitwood, 1938; for review, see Christenson, 1950). Early observations by light microscopists could discern the VL and CL of the outer eggshell, as well as an inner layer, often referred to as the vitelline membrane (see Section 7). We now know this layer to be the lipid-rich permeability barrier. Later, when electron microscopists began looking at the nematode eggshell, their fixation techniques likely destroyed this permeability barrier. The trilaminar outer eggshell survived, and presumably the innermost layer was assumed to be the lipid-rich permeability barrier (S. Olson, personal communication). The recent findings by Olson et al. (2012) have dramatically altered our view of the *C. elegans* eggshell; these new findings strongly suggest that this third layer of the trilaminar outer eggshell is composed of proteoglycans. The two best-characterized proteoglycans in this layer are CPG-1/CEJ-1 and CPG-2 and is thus termed the CPG layer (Figure 1 and Figure 3). This layer is the third layer to be synthesized. Due to the sequential synthesis of the eggshell, perturbations of the CPG layer also result in defects in the synthesis of the fifth layer, the lipid-rich layer (see Section 7). Since it is the lipid-rich layer that makes up the permeability barrier, RNAi conditions or mutants that disrupt the formation of the CPG layer will result in embryos that are permeable due to defects in the subsequent formation of the lipid-rich layer.

### 5.1. Cortical Granules

**Figure 4:**
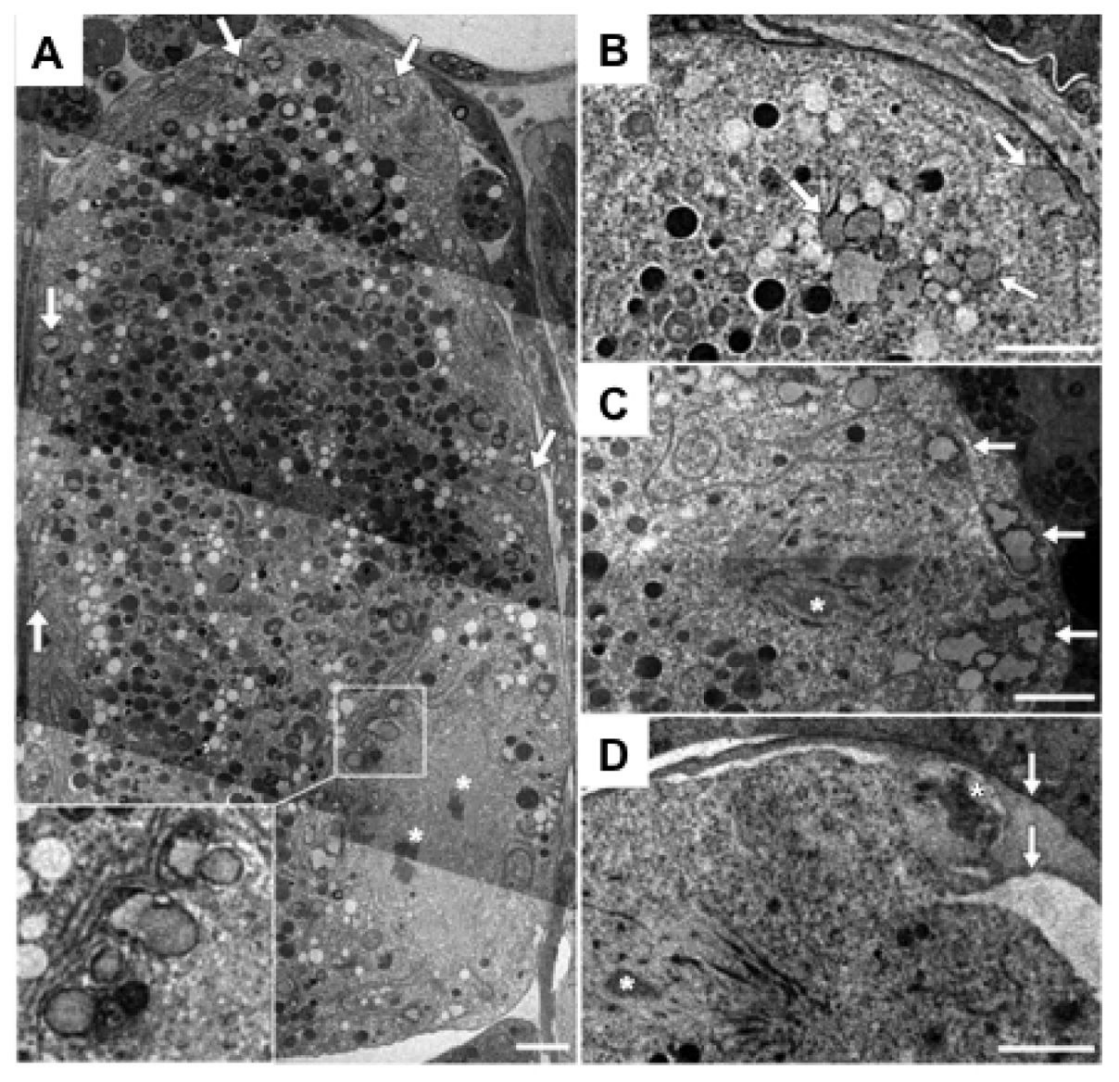
TEM of cortical granules. (A) A mature *C. elegans* oocyte contains cortical granules (arrows); inset shows higher magnification of vesicles near the oocyte chromosomes (asterisks). (B) An embryo within the spermatheca contains a cortical granule near the plasma membrane (upper right arrow), and a cluster of heterogeneous vesicles (lower arrows). (C) A metaphase I embryo contains cortical granules (arrows) distributed across the cortex (asterisk denotes chromosome in spindle). The cortical granules are found in close association with reticulate ER (A–C). (D) Embryos at metaphase II (chromosome in spindle indicated by lower left white asterisk) lack cortical granules, and the polar body (upper right white asterisk) is trapped between eggshell layers (arrows). Scale bar: 2 μm. Figure and legend are from Bembenek et al., 2007. Reprinted with permission from Development.

**Figure 5:**
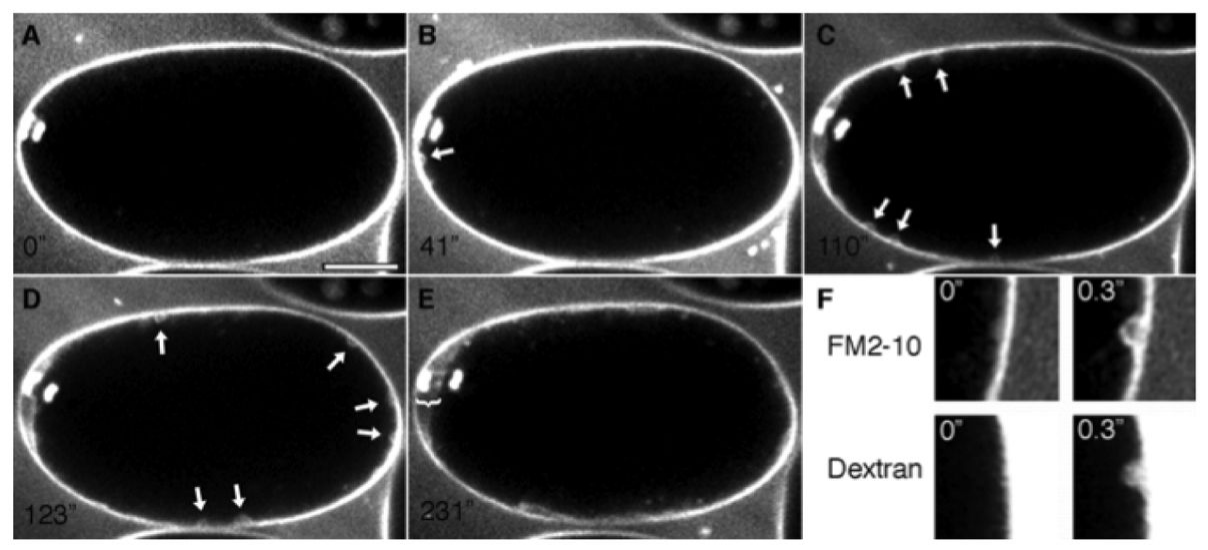
A wave of cortical granule exocytosis during anaphase I. *C. elegans* embryos expressing histone::GFP and labeled with the plasma membrane dye FM2-10 were imaged using a Swept Field Confocal system every 200 milliseconds. After chromosome separation initiated (A), exocytic events (arrows) occurred near the spindle (B) and spread across the cortex (C, D) before the completion of anaphase I. Images in C and D are maximum projection summations of several frames in which a vesicle fusion event occurs. A gap is formed between the plasma membrane and the vitelline layer (now known as the permeability barrier) near the polar body (E, bracket). (F) Images of the plasma membrane before and after exocytosis, labeled with FM2-10 or a fluorescent dextran. Scale bar: 10 μm. Figure and legend are from Bembenek et al., 2007. Reprinted with permission from Development.

The formation of the CPG layer is mediated by the exocytosis of the cortical granules (CGs), which occurs during anaphase of meiosis I (Bembenek et al., 2007) (Figure 4 and Figure 5). CGs have been defined in many metazoans as vesicles that are packaged and stored just underneath the oocyte plasma membrane and released in response to fertilization, with their major function being the modification of the embryonic surface to make it more stable and less penetrant to additional sperm. It is likely that the chitin layer is required to catch the contents of the CGs because embryos that lack the chitin layer but exhibit appropriate CG exocytosis are osmotically-sensitive (presumably because the components of the subsequent layers diffuse away from the embryo and are not assembled as a layer).

Studies with a caveolin homolog, *cav-1,* were the first to demonstrate vesicles in *C. elegans* that had some features reminiscent of classical CGs (Sato et al., 2006). CAV-1 is trafficked in a dynamic manner over the time period of late oogenesis and early embryogenesis. Just prior to ovulation, CAV-1::GFP is found in vesicles clustered around the oocyte nuclear membrane and at the cell periphery. At anaphase I, which occurs after ovulation and fertilization, CAV-1::GFP vesicles move to the cortex and fuse with the plasma membrane in a wave across the zygote to exocytose their contents. CAV-1::GFP is then immediately endocytosed and rapidly degraded. This stereotypical movement and fusion of vesicles in response to fertilization led to the acceptance of these vesicles as the *C. elegans* cortical granules. A *cav-1* deletion mutant, alone or in combination with a *cav-2* mutant, does not produce an eggshell phenotype nor result in embryonic defects (Sato et al., 2008). Thus, caveolin is not essential for CG biogenesis or function, yet is a useful marker of CGs.

In live assays UGTP-1::GFP (a putative Golgi marker) and CAV-1::GFP were used as markers of the CGs to characterize the real-time trafficking of these vesicles (Bembenek et al., 2007). Exocytosis starts at anaphase I, about 90 seconds after the onset of chromosome separation, at the anterior pole of the zygote, and concludes prior to the formation of the first polar body. This live system was used to examine the effect of depletion of several canonical trafficking genes, as well as other genes implicated in eggshell formation, in order to assess if these genes had a role in CG exocytosis. The most severe disruption of CG trafficking results from knocking down the Anaphase Promoting Complex (APC) or other cell cycle regulators, specifically ones that regulate the metaphase-to-anaphase transition. If the cell cycle does not progress through anaphase I no CG fusion and exocytosis occur. The cell cycle protease, separase (SEP-1), localizes to the CGs during anaphase I. When SEP-1 is depleted, CG exocytosis is compromised and the embryos are osmotically defective (Bembenek et al., 2007). This is consistent with the observed cell-cycle dependent precocious trafficking of CGs in *wee-1.3* RNAi-treated animals, which undergo oocyte maturation prematurely (Bembenek et al., 2007).

Depletion of CHS-1 did not alter vesicle trafficking, supporting the model that the CL and CPG layers are formed by two independent pathways. This is further supported by immunoEM studies that show the CL and CPG layers as two distinct layers (Figure 2 and Figure 3) (Olson et al., 2012). Depletion of CPG-1/2 or SQV-4 (proteins involved in proteoglycan synthesis) however does change the size of the vesicles, suggesting that these genes regulate CG content (Bembenek et al., 2007).

Ultrastructural analysis of putative CGs in *Ascaris lumbricoides* showed the presence of vesicles one micron in size with an unusual morphology reminiscent of refringent granules (Foor, 1967). The granules are associated with the ER in the oocyte, move to the cortex during prometaphase, and then disappear by anaphase of meiosis I, consistent with the exocytosis observed at this stage of the cell cycle with CAV-1 vesicles. Definitive demonstration that these vesicles are CGs will require a more rigorous immunoEM approach, with the aim of positively identifying some of the putative CG markers such as CAV-1, UGTP-1 or the lectin, WGA.

How do CGs fuse with the plasma membrane to release their cargo? Depletion of canonical trafficking proteins inhibited vesicle fusion. As mentioned earlier, depletion of the vesicle transport Rab GTPase, RAB-11-1, affects CG exocytosis (but not CG formation): CG vesicles remain clustered around the nucleus and do not move to the cell periphery where fusion with the plasma membrane occurs. The CGs also persisted in the embryos long after fertilization (Sato et al., 2008). The SNARE proteins regulate the docking and fusion of vesicles with their target membranes; vesicles have v-SNAREs and target membranes have t-SNAREs. Depletion of SYN-4/SYX-4 (a syntaxin-related t-SNARE) (Jantsch-Plunger and Glotzer, 1999) resulted in a very similar phenotype to that of RAB-11.1 depletion (Sato et al., 2008). The terminal phenotype of both RNAi depletions is osmotically sensitive, multinucleate one-cell embryos that are defective for cytokinesis (Sato et al., 2008; Sonnichsen et al., 2005). As might be expected, GFP::RAB-11.1 localizes to CGs in the −1 oocyte and +1 zygote only, shortly before the exocytosis of these vesicles.

While depletion of genes involved in proteoglycan synthesis (*sqv-5*) or CG exocytosis (APC genes) produces orderly and oval shaped embryos, depletion of RAB-11.1 and SYN-4/SYX-4 yields irregularly-shaped embryos that seem to lack all mechanical support, which is unexpected due to the persistence of chitin staining in the RAB-11.1-depleted embryos (Sato et al., 2008). This observation may indicate that the modifications of the CL that occur in embryos after the completion of the meiotic divisions are essential for the structural integrity of the eggshell or may reflect the earlier roles these genes play in germline and oocyte development (Green et al., 2011). These data suggest that RAB-11.1-dependent vesicle transport and fusion and APC-dependent CG exocytosis are integral to the integrity of the CL and CPG layers of the eggshell.

### 5.2. Components of the cortical granules

**Figure 6:**
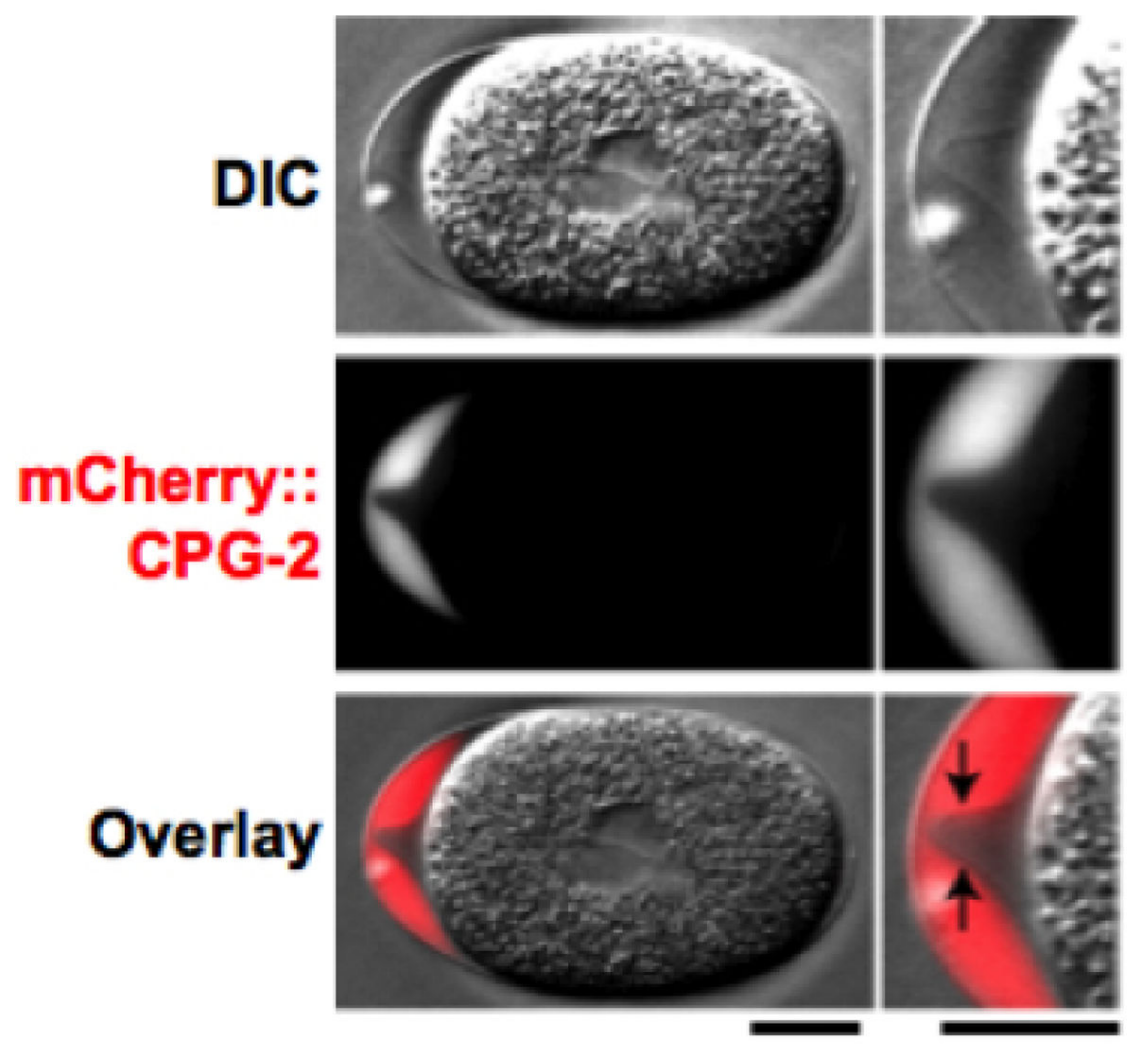
CPG-2::GFP is detected in the extra-embryonic matrix. DIC (top) and fluorescence (middle; red in merge) images of a one cellstage mitotic embryo expressing mCherry::CPG-2 (n = 9). The embryo anterior is magnified on the right. The contrast of the top DIC image has been adjusted to visualize the edge of the permeability barrier (indicated by the black arrows in the merge). Bars, 10 μm. Figure and legend are from Olson et al., 2012. Reprinted with permission from The Journal of Cell Biology.

The cargo of the CGs is likely to contain components necessary to complete the eggshell structure as well as provide the material for the layer between the embryo and the eggshell (a.k.a. the extra-embryonic matrix, see Section 6). In a screen for vulval defects, the eight *sqv* (squashed vulva) genes were isolated (Herman et al., 1999) and all were shown to be in the chondroitin and heparan sulfate synthesis pathways, which produce proteoglycans (Hwang et al., 2003a; Hwang et al., 2003b; Hwang and Horvitz, 2002; Berninsone et al., 2001; Herman et al., 1999; Herman and Horvitz, 1999). Proteins in this class are often found in hydrated extracellular matrix environments. Chondroitin, a glycosaminoglycan, is added to a protein core in a stepwise process by a series of synthetic enzymes with chondroitin synthase (SQV-5) at the end of the synthetic pathway (Hwang et al., 2003b). In addition to vulval defects, mutations in these genes result in embryonic lethality. Immunolocalization studies demonstrated that SQV-5 is localized to punctate foci in oocytes that are likely to reflect localization to the Golgi, as observed for such enzymes in other organisms (Hwang et al., 2003b; Mizuguchi et al., 2003; Hwang and Horvitz, 2002). Anti-chondroitin antibodies stain ring-shaped structures that co-localize with CAV-1::GFP, indicating that chondroitin is a cargo of the CGs. Chondroitin localizes to CAV-1 bodies just prior to fertilization (Sato et al., 2008) and then localizes to the eggshell (Olson et al., 2012). CPG-1 and CPG-2 are the protein cores of two (of nine) chondroitin proteoglycans identified biochemically in *C. elegans* (Olson et al., 2006). These protein cores have multiple putative attachment sites for a chondroitin chain (Figure 3). CPG-1::GFP and CPG-2::GFP are secreted from caveolin-enriched CGs during meiosis I; immunoEM shows that they associate with the innermost layer of the trilaminar eggshell (Figure 3) (Olson et al., 2012). CPG-1::GFP has a punctate cortical localization in oocytes and then is gradually enriched in a smooth, evenly-distributed pattern in the CPG layer of the eggshell. CPG-2::GFP is detected in the extra-embryonic matrix (Olson et al., 2012) (see Section 6 and Figure 6).

Depletion of genes in the chondroitin proteoglycan pathway causes defects consistent with a role for these proteins in eggshell synthesis. When CPG-1/2 or SQV-5 are depleted by RNAi, global defects are observed at the one-cell stage and embryonic lethality results (Olson et al., 2006; Lee and Schedl, 2001; Herman et al., 1999). Embryos exhibit osmotic sensitivity and fail to complete several early developmental events, including polar body extrusion and membrane ruffling. In addition, embryos fail to form the extra-embryonic matrix (see Section 6). Furthermore, cytokinesis was not observed. Curiously, *sqv* embryos were reported to retain their shapes better than unfertilized oocytes (even after sodium hypochlorite treatment to remove the VL) (Herman et al., 1999), indicating that these embryos may have partial eggshells, as would be expected if the CL was unaffected. In order to determine whether the eggshell was the primary source of defects in these embryos, the embryos were cultured in an isotonic environment. The early embryonic defects of SQV-5-depleted embryos, which still retain the trilaminar outer eggshell, were rescued by osmotic support media. The same treatment only partially rescued CPG-1/2-depleted embryos. EM of embryos co-depleted of CPG-1/2 revealed the absence of the CPG layer (Olson et al., 2012). Deletion mutants of either *cpg-1* or *cpg-2* alone appear to be homozygous viable (http://www.wormbase.org/), indicating that these genes are probably redundant. These observations imply that the core proteins of the chondroitin proteoglycans are necessary for eggshell function, while the chondroitin modifications themselves are not necessary for the synthesis of the CPG layer. This work suggests that the protein cores have a function separable from the chondroitin chains alone.

## 6. Layer four: the extra-embryonic matrix

**Figure 7:**
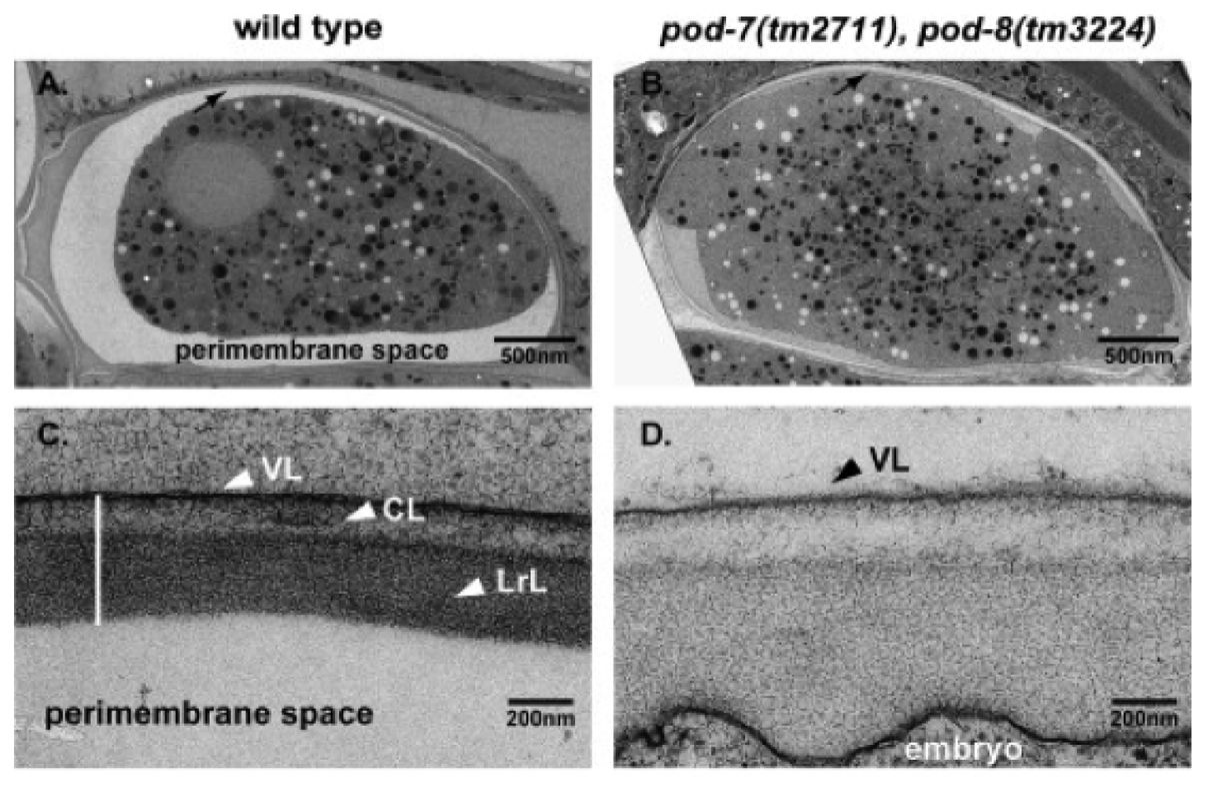
The extra-embryonic matrix (which is also known as the perimembrane space or perivitelline space). (A, B) EM low magnification (A, wild-type; B, *pod-7(tm2711) pod-8(tm3224)*), note the swollen embryo in B; arrows point to the eggshell region. (C, D) EM high magnification (C, wild-type; D, *pod-7(tm2711) pod-8(tm3224)*). Wild-type embryo shows a typical, well-separated three-layered structure of the eggshell; VL, vitelline layer; CL, chitin layer; LrL, lipid-rich layer (which is now called the CPG layer). *pod-7(tm2711) pod-8(tm3224)* display a disorganized arrangement of the layers. There is no clearly distinguishable LrL. Figure and legend are from Benenati et al., 2009. Reprinted with permission from Mechanisms of Development.

There is a fluid-filled layer that separates the trilaminar outer eggshell from the permeability barrier (see Section 7) and embryo. This layer has previously been called the perivitelline space, perimembrane space, or the extra-embryonic space. See Figure 7 for a nice example of this layer (Benenati et al., 2009). Historically, because the permeability barrier is visible by light microscopy, it was called the vitelline membrane, and the space surrounding this membrane was called the perivitelline space. Since the terminology of vitelline membrane is no longer in use, this fluid-filled layer is now called the extra-embryonic matrix (EEM) (Johnston and Dennis, 2012). This layer is most obvious when observed at the anterior end of young embryos where there is a lot of space between the eggshell and the permeability barrier of the embryo. This layer is much less obvious along the dorsal or ventral surfaces of the embryo. It is thought that this layer is fluid-filled and contains a number of proteins (Olson et al., 2012; Johnston et al., 2006). The CPG-2::mCherry protein, for example, is an excellent marker for the extra-embryonic matrix (Figure 6) (Olson et al., 2012). Defects in the synthesis of chondroitin account for abnormalities in the synthesis of this layer, as evident from the analysis of the *sqv* mutants (Hwang and Horvitz, 2002). Without this layer, there is no separation between the plasma membrane of the embryo and the trilaminar outer eggshell; this defect likely accounts for the cytokinesis failures in all *sqv* (and *cpg*) mutants. It is thought that the plasma membrane and eggshell adhere to each other in the absence of this layer (as observed when CPG-1 and CPG-2 are co-depleted), thus contributing to these cytokinesis failures (Olson et al., 2012).

## 7. Layers five and six: the permeability barrier and the peri-embryonic layer

The permeability barrier is the layer of the eggshell that is responsible for the osmotic integrity of the embryo. It serves as a permeability barrier to prevent large molecules or toxins in the external environment from penetrating into the embryo. It has been shown that embryos can develop normally (in osmotic support media) if both the VL and CL (and presumably the CPG layer) are removed, leaving just the permeability barrier to protect the embryo (Schierenberg and Junkersdorf, 1992).

**Figure 8:**
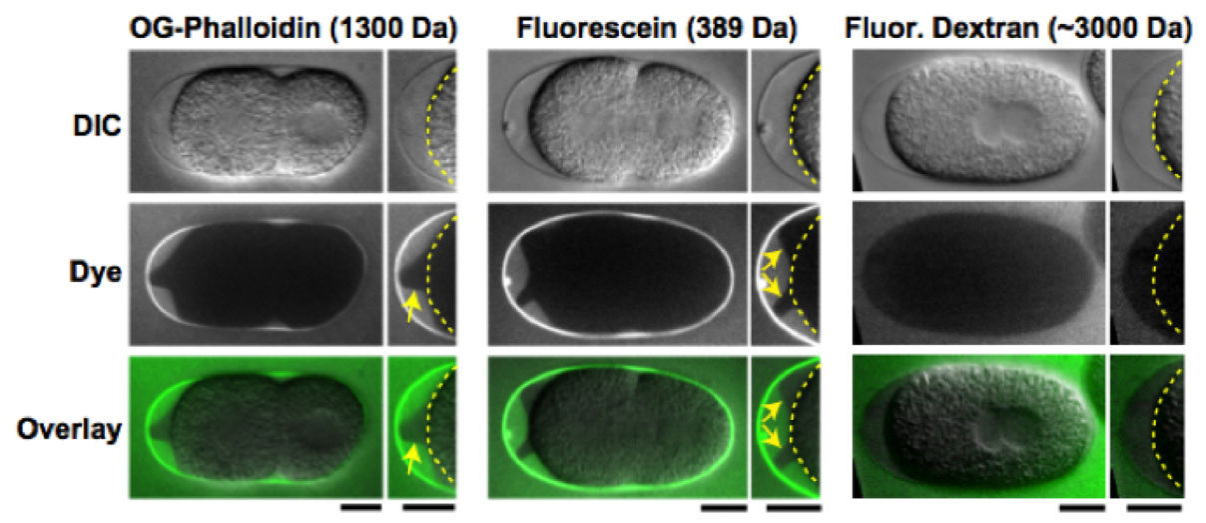
The permeability barrier is a distinct envelope that forms between the trilaminar eggshell and the embryo. (A) Embryos placed in Oregon green (OG) phalloidin (left; n = 14), fluorescein (middle; n = 7), or 3,000-D fluorescein dextran (right; n = 10) were imaged by differential interference contrast (DIC; top row) and fluorescence (middle row; green in merge) microscopy. Magnified views of the embryo anterior are shown on the right (yellow dashed lines mark the embryo plasma membrane; yellow arrows point to the edge of the permeability barrier). Bars, 10 μm. Figure and legend are from Olson et al., 2012. Reprinted with permission from The Journal of Cell Biology.

**Figure 9:**
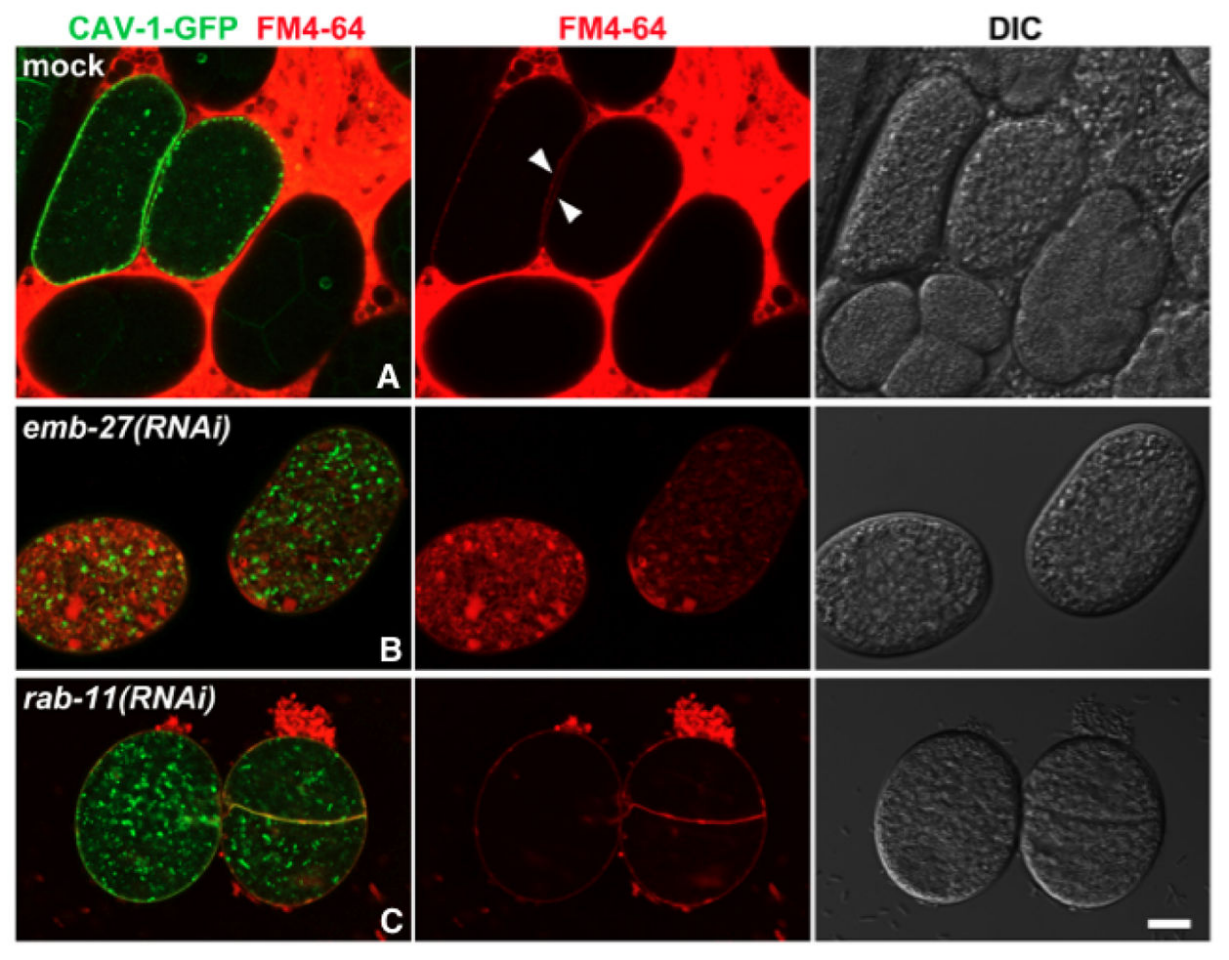
Dye assay to monitor the permeability barrier. The permeability of embryos to FM4-64 was examined in mock (A), *emb-27(RNAi)* (B) and *rab-11.1(RNAi)* (C) animals expressing CAV-1::GFP. Newly fertilized wild-type embryos are permeable to FM4-64 (A, arrowheads indicate the plasma membrane stained with FM4-64) but blastomeres are not (embryos on the bottom and to the right in A). *emb-27* is a gene that codes for the CDC16 subunit of the Anaphase Promoting Complex, depletion of which disrupts the formation of the permeability barrier. The *emb-27(RNAi)* and *rab-11.1(RNAi)* embryos are permeable to FM4-64. Nomarski image is on the far right in each row. Scale bar: 10 μm. The data for this figure was reproduced by Miyuki Sato from her 2008 paper (Sato et al., 2008).

Dyes such as OG-phalloidin, fluorescein, and fluorescein dextran have been used to help visualize the permeability barrier (Figure 8) (Olson et al., 2012). A useful assay to test the integrity of this barrier is to soak embryos in the lipophilic dye FM4-64. Wild-type embryos that have completed the meiotic divisions are impermeable to this dye, whereas zygotes arrested in metaphase I are permeable (Sato et al., 2008; Johnston et al., 2006; Rappleye et al., 1999). This dye will be incorporated into the plasma membrane of the embryo if no permeability barrier exists (Figure 9). Embryos depleted of RAB-11.1 (see Section 5) were permeable to this dye, further suggesting that the cargo of the CGs is responsible for indirectly establishing the permeability barrier (Sato et al., 2008).

**Figure 10:**
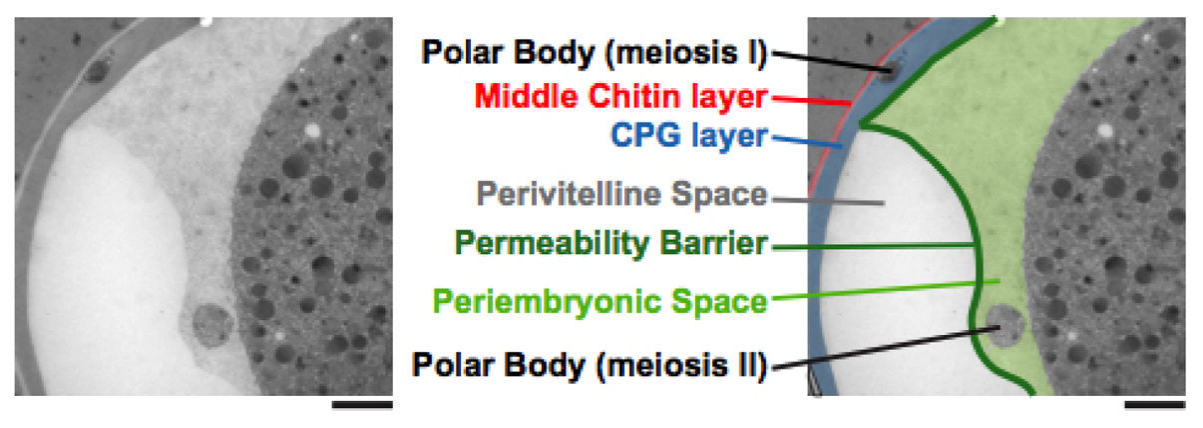
The permeability barrier encloses the second polar body. Transmission electron micrographs of a control embryo showing the location of the polar bodies extruded during anaphase of meiosis I (embedded in the CPG layer) and during anaphase of meiosis II (in the peri-embryonic layer). Bar, 1 μm. Figure and legend are from Olson et al., 2012. Reprinted with permission from The Journal of Cell Biology. The perivitelline space is called the extra-embryonic matrix and the peri-embryonic space is called the peri-embryoninc layer throughout this chapter.

**Figure 11:**
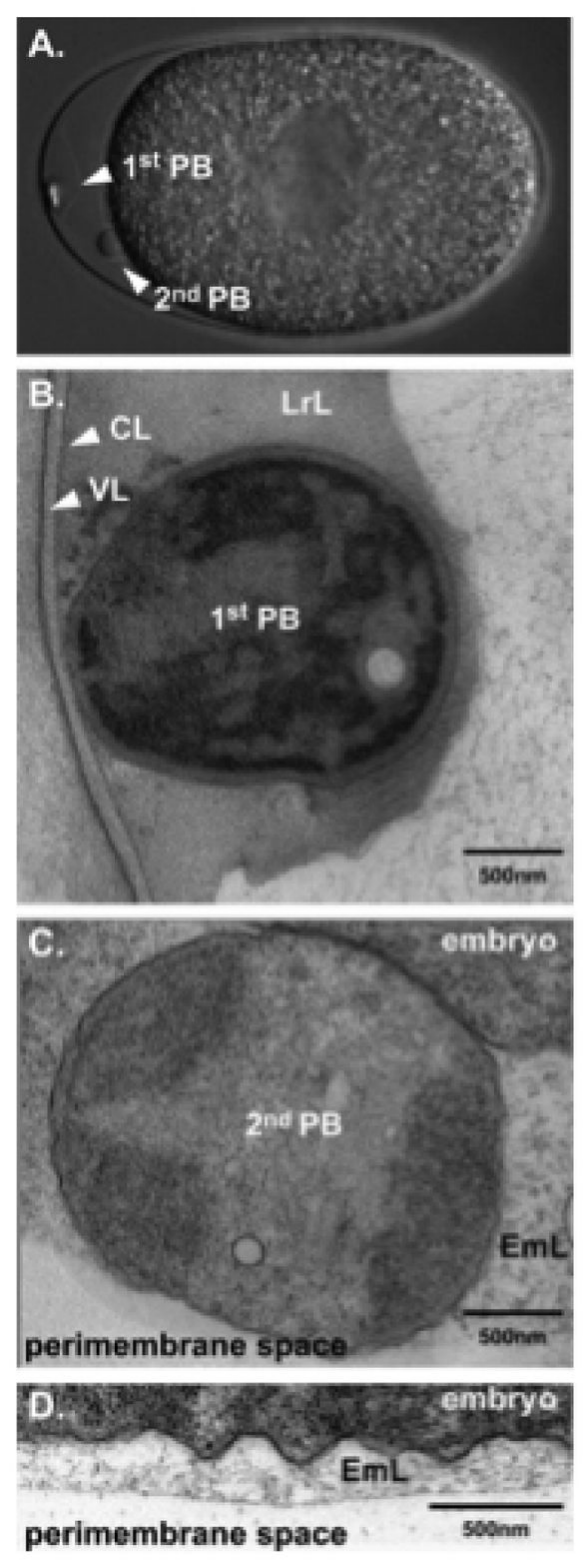
The two polar bodies in *C. elegans* embryos are differently localized after their extrusion. (A) DIC image of a wild-type embryo with two visible polar bodies. The first polar body is localized at the apex of the anterior whereas the second polar body stays in contact with the plasma membrane. (B) and (C), EM of sections. (B) The first polar body is tightly associated with the chitin layer and is fully embedded in the LrL (which is now called the CPG layer). (C) The second polar body is separated from the embryo and is surrounded by a filamentous, embryonic layer (EmL, which is now referred to as the peri-embryonic layer) (D) The entire embryo is surrounded by the EmL or peri-embryonic layer. Figure and legend are from Benenati et al., 2009. Reprinted with permission from Mechanisms of Development. The perimembrane space is called the extra-embryonic matrix throughout this chapter.

**Figure 12:**
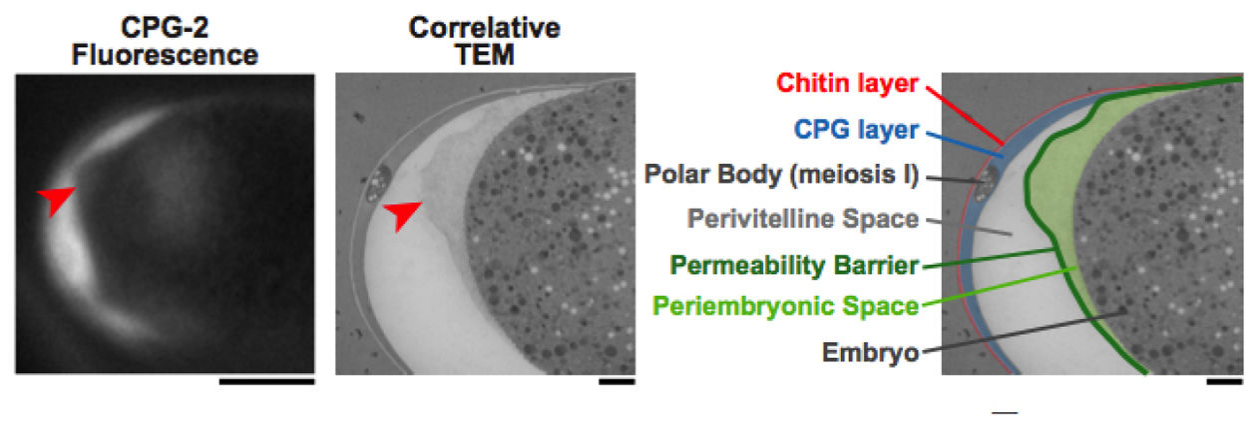
The peri-embryonic space (layer) is quite fluid and amorphous. Left: A fluorescence image of CPG-2 was acquired immediately before cryoimmobilization and processing for transmission electron microscopy (n = 3). Middle: Alignment of the resulting correlative transmission electron micrograph (TEM) image allows visualization of the edge of the permeability barrier (red arrowheads). Right: A pseudocolored micrograph illustrates the location of the chitin and CPG eggshell layers along with the edge of the permeability barrier. The peri-embryonic layer and perivitelline space (extra-embryonic matrix) and the first polar body are also labeled. Bars, 10 μm (left); 1 μm (middle and right). Figure and legend are from Olson et al., 2012. Reprinted with permission from The Journal of Cell Biology.

This fifth layer has only recently been identified as the permeability barrier; it formerly was known as the vitelline membrane (Chitwood, 1938; Bird, 1971), a structure visible by DIC microscopy that surrounds the embryo proper (whose boundary is defined by its plasma membrane). It is separated from the trilaminar outer eggshell by the extra-embryonic matrix. The permeability barrier encloses the second polar body (Figure 1, Figure 10, and Figure 11). The definition of the permeability barrier differs in two recent publications (Olson et al., 2012; Benenati et al., 2009). The definition by Benenati *et al*. (2009) described a layer that completely surrounds the embryo and is approximately 100–200 nm thick (Benenati et al., 2009). While Benenati et al. refer to this as a single layer, Olson et al. (2012) consider this as two distinct layers. They describe the lipid-rich permeability barrier as a thin membrane-like structure (Olson et al., 2012), distinct from the fluid-filled layer that lies beneath it [which they call the peri-embryonic space (Figure 1, Figure 10, and Figure 11) and Benenati et al. refer to as a filamentous embryonic layer (Benenati et al., 2009)]. This layer also provides protection for the embryo and directly apposes the embryo proper. This layer appears to be quite fluid and amorphous (Figure 12) and likely accounts for the high mobility of the second polar body during early embryonic development. It is likely that novel processing and fixation conditions are responsible for the visualization of this previously unnoticed layer. We propose calling this sixth layer the peri-embryonic layer, rather than peri-embryonic space (Olson et al., 2012) or embryonic layer (Benenati et al., 2009). We also agree that the permeability barrier should be considered a separate layer of the eggshell.

Recent studies have shown that formation of the permeability barrier requires passage through anaphase of meiosis II (Olson et al., 2012). This timing is distinct from the timing of the formation of the trilaminar eggshell, which is established by anaphase of meiosis I. Historically, the permeability barrier was thought to be the innermost layer of the trilaminar eggshell and was thought to be lipid-rich. However, as stated earlier, studies by Olson et al. (2012) revealed that the permeability barrier was a layer separated from the trilaminar eggshell by the extra-embryonic matrix. Since most studies prior to 2009 did not observe this fifth inner layer in fixed samples, and since embryos stripped of the VL and CL were still impermeable (Schierenberg and Junkersdorf, 1992), it had been assumed that the third layer was the permeability barrier. Given the most recent studies (Olson et al., 2012), it now appears that the lipid-rich layer responsible for protecting the embryo apposes the embryo and is not part of the trilaminar outer eggshell.

Phenotypes formerly attributed to the third layer may need to be re-evaluated to determine whether it was really the CPG layer or the lipid-rich permeability barrier that was defective. Either way, it appears that the lipid-rich layer has consistently been assigned the function of permeability barrier. It is the positioning of this layer that has recently been more clearly demonstrated (Olson et al., 2012). Since the fifth layer had not been observed before in fixed embryos, it is difficult to know how many previous studies affected this layer. For example, how do defects in CG exocytosis affect the formation and structure of both the CPG layer and the lipid-rich permeability barrier? The observation that defects in CG exocytosis are correlated with osmotic integrity defects suggests that both the CPG layer and the permeability barrier must be compromised. Lacking in most of the earlier studies are EM images to determine the integrity of all the layers of the eggshell.

Evidence that the permeability barrier is a lipid-rich layer came from earlier studies in which the depletion of lipid biosynthetic enzymes caused the OID phenotype (Benenati et al., 2009; Rappleye et al., 2003; Tagawa et al., 2001) (Figure 7). *pod-2* was recovered in a genetic screen for mutants with polarity and osmotic defects (Section 8) and encodes an acetyl-CoA carboxylase ortholog (Rappleye et al., 2003). Two other lipid regulators were discovered in the same class: *emb-8*, which encodes the NADPH cytochrome P450 reductase, an enzyme that catalyzes the modification of fatty acids, and *fasn-1*, a fatty acid synthase (Rappleye et al., 2003). It was demonstrated that the addition of specific fatty acids to the diet of *pod-2* mutant animals could rescue the polarity phenotypes, but not the osmotic phenotype (Rappleye et al., 2003). However, embryos depleted of EMB-8 and POD-7/8 (cytochrome P450 family members involved in the hydroxylation of lipids) were completely rescued by a diet of *C. elegans* lipid extracts (Benenati et al., 2009). These experiments together suggest that different lipids may be responsible for the polarity defects versus the osmotic defects. Two other proteins recently identified, PERM-1 and DGTR-1, suggest that ascaroside glycolipids may be an essential component of the permeability barrier (Olson et al., 2012). Determining the specific lipid and protein components of this fifth layer will certainly shed light on how this layer protects the embryo.

## 8. Does actin play a role in eggshell formation?

It has been observed that an actin cap forms on the posterior cortex of the oocyte several minutes after its entry into the spermatheca (Maruyama et al., 2007). This F-actin cap is thought to mark the site of sperm entry. In embryos depleted of EGG-3 or EGG-4/5, this cap is not restricted to the posterior cortex and disperses aberrantly (Parry et al., 2009; Maruyama et al., 2007). Most other eggshell studies have not assayed for this actin cap and thus its role in the initiation of eggshell formation remains unclear. Currently this cap remains a useful marker of the sperm entry site. At the anterior pole of the embryo, actin is required for polar body extrusion (Velarde et al., 2007). Severe RNAi depletion of F-actin not only disrupts polar body extrusion, but also results in cytokinesis defects during the mitotic divisions (Velarde et al., 2007).

**Figure 13:**
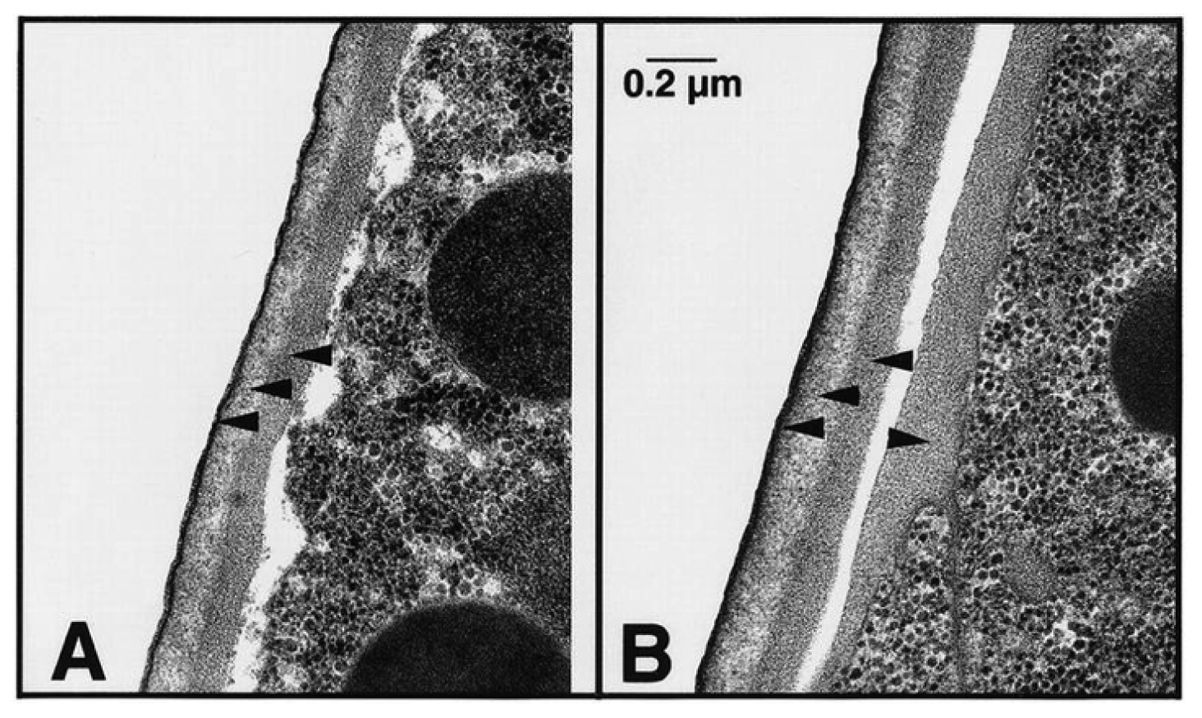
*pod-*1 mutants have a normal trilaminar eggshell but an abnormal peri-embryonic layer. Shown is the ultrastructure of wild-type and *pod-1* mutant eggshells. In each panel, an embryo with its eggshell is shown. (A) Three layers are visible in the wild-type *C. elegans* eggshells: a thin vitelline layer (black line indicated with lower arrowhead), a chitinous layer (light-staining layer indicated with central arrowhead), and a lipid-rich layer (darker layer indicated by upper arrowhead; now known as the CPG layer). (B) All three layers are present and appear normal at this resolution in the *pod-1(ye11)* eggshell (left pointing arrowheads). However, an extra layer of dark-staining material is evident on the outside of the *pod-1* mutant embryos (right pointing arrowhead) that is not evident in wild type. Figure and legend are from Rappleye et al., 1999. Reprinted with permission from Genes and Development.

The Aroian laboratory conducted a screen to identify a Polarity and Osmotic Defective (*pod*) class of mutants whose embryos develop with osmotic sensitivity and polarity defects (Rappleye et al., 1999). POD-1 is a coronin-like protein, an actin-binding protein that localizes to the anterior cortex of one-cell embryos. Embryos lacking POD-1 fail to extrude polar bodies, swell in hypotonic buffer and shrink in hypertonic buffer, and are permeable to fluorescent dyes (Rappleye et al., 1999). These embryos also display polarity defects, however, the polarity defects are separable from the osmotic defects (Rappleye et al., 1999). EM analysis of the eggshells of wild-type and mutant embryos revealed that *pod-1* mutants have a normal trilaminar outer eggshell but also possess an aberrant extra layer that surrounds the embryo proper (Figure 13). Olson et al. (2012) proposed that this extra layer was a second CPG layer that resulted from delayed CG exocytosis. How an actin-binding protein functions in the osmotic integrity of the embryo awaits further investigation.

The POD-1 studies discussed above were the first to suggest a connection between osmoregulation and the actin cytoskeleton. Defects in osmoregulation can certainly perturb the actin cytoskeleton and cytokinesis. However, the reverse has not been observed: studies of actin RNAi or inhibition have not described defects in the osmotic sensitivity or permeability of the early embryo.

Another link to the actin cytoskeleton and osmoregulation came from studies of the *cyk-*3 mutant. *cyk-3* encodes a ubiquitin C-terminal hydrolase. Depletion of this gene results in oval, osmotically sensitive, dye-permeable embryos. In addition, polar bodies are not extruded, no membrane contractions are observed, and pseudocleavage and cytokinesis fail (Kaitna et al., 2002). Actin distribution in the embryonic cortex is also disrupted. The authors observed a partial rescue of the cytokinesis defects using osmotic support media. Vesicle trafficking in these mutants has not yet been investigated. Presumably there are defects as the dye permeability of the embryos suggests the improper formation of the permeability barrier. The authors hypothesize that the target of this hydrolase is a protein that controls osmolarity, such as an ion channel.

## 9. The block to polyspermy

The block to polyspermy is a mechanism present in all sexually reproducing organisms to ensure that progeny have a euploid genome. There are typically two levels of block: a fast block at the membrane level that is based on membrane potential, and a slow block that generally involves the modification or generation of an extracellular matrix that acts as a physical barrier to sperm entry (Wong and Wessel, 2006). There is currently no evidence in *C. elegans* that a disruption of membrane potential in the fertilized embryo leads to a polyspermic phenotype. Furthermore, no mutants or RNAi depletion studies with ion channels have resulted in polyspermy. The only observations of polyspermy have come from RNAi and mutant studies on genes that contribute to the synthesis of the chitin layer of the eggshell. The synthesis of a chitinous eggshell is likely the slow block mechanism in *C. elegans*. Johnston et al. (2010) recently reported that CHS-1, GNA-2, and CBD-1 depletions lead to polyspermy. *egg-4/5* double mutants are also polyspermic (Parry et al., 2009). Johnston et al. (2010) also assert that embryos depleted of SPE-11, EGG-1, EGG-2, and EGG-3 are fertilized by more than a single sperm. In contrast, Kadandale et al. (2005) had previously shown that depletion of EGG-1/2 by RNAi led to infertility (no gamete fusion). Currently there is no good explanation for the discrepancy between these reports. Interestingly, most polyspermic embryos have just two sperm pronuclei and the authors suggest that there must be a second, non-chitin-dependent block (Johnston et al., 2010).

In contrast to the polyspermy exhibited in the Johnston study (2010) and the *egg-4/5* study (Parry et al., 2009), there is at least one RNAi condition in which oocytes appear to be refractive to fertilization (Allen et al., 2014; Burrows et al., 2006). The Wee1 kinases are responsible for the phosphorylation and inhibition of Cdk1 in eukaryotes. Of the two Wee1 kinase orthologs in *C. elegans*, WEE-1.3 is expressed in the germline (but not exclusively) and is required to delay maturation of the oocyte until it is in the −1 position in the oviduct (Allen et al., 2014; Burrows et al., 2006). When WEE-1.3 is depleted, oocytes precociously demonstrate hallmarks of maturation such as phospho-histone H3 antibody staining and nucleolar breakdown. Interestingly, these oocytes pass through a sperm-filled spermatheca yet do not get fertilized. Is this due to a premature block to polyspermy or a premature CG movement to the cortex (Bembenek et al., 2007)? And, if so, does this indicate that the polyspermy block is coupled to the cell cycle? Depletion of WEE-1.3 could precociously activate the cell cycle, resulting in the premature internalization of the EGG proteins and MBK-2, and thus prevent fertilization. Further studies of this rare phenotype will hopefully uncover some additional players in the block to polyspermy.

**Figure 14:**
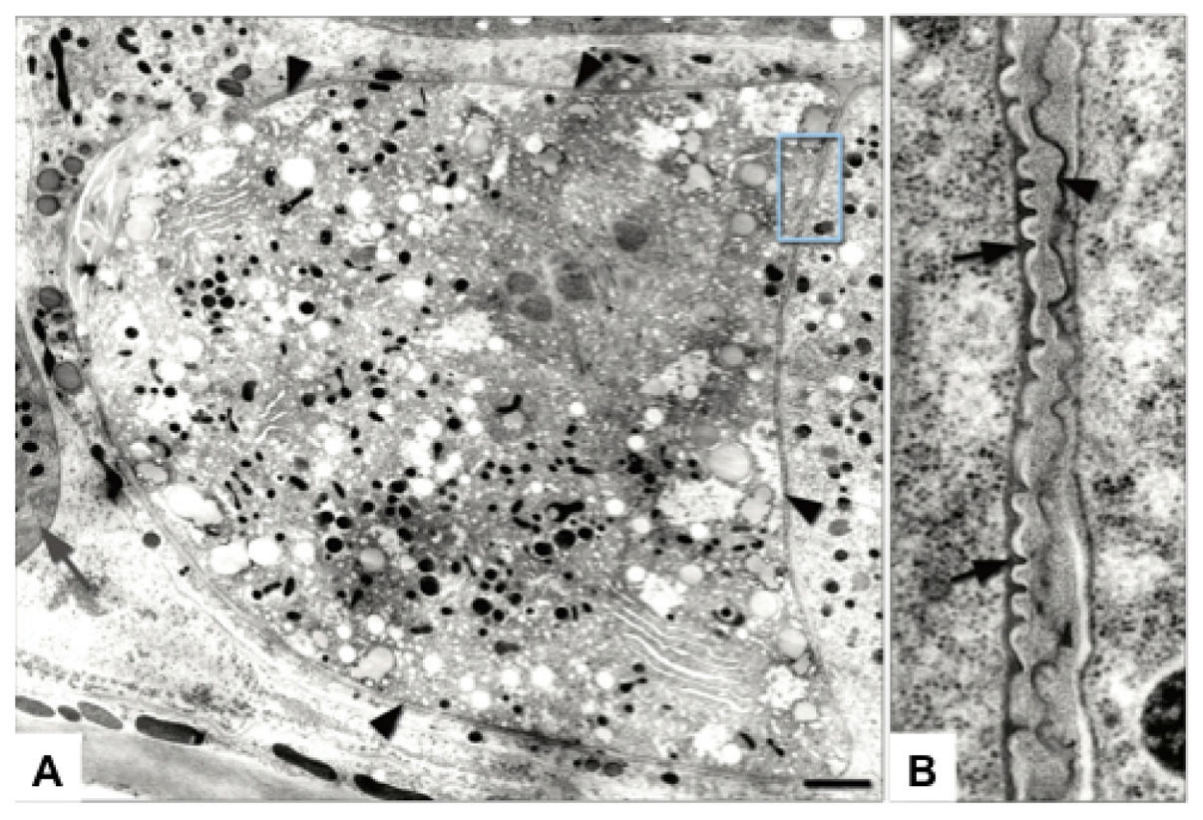
Newly fertilized embryos are covered with a crenelated membrane. (A) For a brief period, a recently fertilized embryo becomes covered with a dense, crenelated membrane (arrowheads) that may prevent polyspermy. (Gray arrow) Spermatheca. TEM-high pressure freeze fixation, transverse section. Bar, 1 μm. (Image source: [Hall] CL2099-2A.) (B) Same image as in A, magnified. The fertilization membrane of the embryo on the left (arrows) is still attached to its surface whereas that of an older embryo on the right is being sloughed off (arrowhead). Figure and legend are from Hall and Altun, 2008. Reprinted with permission from Cold Spring Harbor Press and David Hall.

One suggestion of a mechanism for the block to polyspermy originates with what has been called a post-ovulatory envelope (David Greenstein, personal communication) or a fertilization membrane (Hall and Altun, 2008). In some TEM images, it appears that unfertilized oocytes and newly fertilized zygotes possess a thin crenellated covering on top of the vitelline layer (David Greenstein, personal communication) (Hall and Altun, 2008) (Figure 14). It is tempting to speculate that this envelope is briefly assembled on all oocytes that pass through the spermatheca (whether they are fertilized or not). This envelope must be added in the spermatheca or uterus. This so-called uterine layer has been observed for numerous nematode species (Foor, 1967). We thus propose, in most situations where sperm-oocyte fusion has occurred, the post-ovulatory envelope or fertilization membrane is disassembled following the synthesis of the eggshell. However, perhaps in situations where the oocytes are not fertilized (such as *spe-9* mutants and feminizing mutants), the disassembly of this post-ovulatory envelope does not occur and remains to stably protect the unfertilized oocyte. This hypothesis is actually borne out by our conversations with David Greenstein, his observations of *spe-8* oocytes, and the images presented by David Hall in the Worm Atlas (Hall and Altun, 2008). If a transient post-fertilization envelope does exist in *C. elegans*, it would help to explain a perplexing observation about the differential stability of unfertilized oocytes and fertilized 1-cell zygotes that have not progressed through their meiotic divisions. Unfertilized oocytes, whether from a *spe* mutant or from a hermaphrodite that has exhausted its sperm, appear round and stable on growth plates. Although they do not possess a chitinous eggshell, they remain intact in the process of going through two spermathecal valves and then the vulva to be deposited externally. Yet *chs-1, gna-2,* and *spe-11* embryos are extremely fragile and often crushed by the time they are laid on the growth plate. Perhaps fertilization activates a process that removes this protective post-ovulatory envelope. But because *chs-1, gna-2,* and *spe-11* embryos are defective in eggshell synthesis, these mutant embryos are subsequently crushed by the forces of egg laying. Thus it has been proposed that this post-ovulatory envelope is responsible for the structural integrity of unfertilized oocytes, and, in fertilized embryos, serves as a block to polyspermy while the chitin layer of the eggshell is being synthesized (Hall and Altun, 2008).

## 10. Screens for eggshell synthesis genes

It is likely that there are several more important players in the process of eggshell formation in *C. elegans*. How will these genes be discovered? Many of the genes suspected of being involved in eggshell synthesis (directly or indirectly) were originally identified in genome-wide RNAi screens (Sonnichsen et al., 2005). These lists of genes with putative eggshell phenotypes should be examined and studied in greater detail. The key phenotypic classes include Osmotic Integrity Defective or OID, and passage through meiosis defective (Sonnichsen et al., 2005). The OID class consisted of 109 genes and has since been expanded by additional screens. Depletion by RNAi of other genes that result in passage through meiosis defects also cause osmotic defects. It is now thought that these eggshell defects are an indirect result of post-fertilization meiotic defects (such as the APC mutants). All of these genes are listed in Table 1 (Section 12). In a recent screen to find genes that, when depleted by RNAi, cause early embryos to be more permeable for drug and small molecule uptake Carvalho et al. (2011), identified a set of 310 candidate genes that, by bioinformatics criteria, might play a role in eggshell synthesis. Of these, 20 resulted in >75% eggshell permeability. These genes had already been identified as members of the OID class of genes, except for *inx-*9, which is included in Table 1.

## 11. Future

The future for the study of the *C. elegans* eggshell remains bright. There are numerous questions that demand investigation. For example, what are the cortical granules components? Knowing the makeup of these granules will be very informative with regard to the fusion of the vesicles to the plasma membrane and the proteins that are delivered to the extracellular environment of the embryo. Likewise, identifying the components of the permeability barrier and the biosynthetic pathways responsible for this structure is key to our understanding of this barrier.

To more adequately follow the synthesis of the various eggshell layers, the field needs more markers for each of the layers. Besides our ability to detect the chitin layer and CPG-1/2, we have few available reagents to label the various eggshell layers. Missing in most studies of the eggshell are EM and immunoEM images of various mutants. Such studies would benefit from images at different stages of early embryonic development to observe the sequential synthesis of the eggshell and the modifications thereof. The observation that most OID embryos also show defects in polar body extrusion and cytokinesis warrants further investigations as to the connection between these phenotypes.

Another important question to address is whether there is a fast block to polyspermy and what role, if any, does the calcium wave play in this block? Few existing mutants or RNAi depletion experiments have resulted in polyspermy, and in those cases, none of the genes yet suggest a clear fast block mechanism. Perhaps a forward genetic screen aimed specifically at identifying polyspermic embryos would help answer this important question.

Is there a sperm component required for CHS-1 activation? Intriguingly, there is evidence from other organisms that chitin synthase needs to be proteolytically processed in order to become functional. In the context of *C. elegans* fertilization, it is tempting to speculate that this regulatory mechanism works via a protease carried by the sperm, which is introduced into the oocyte at fertilization. This mode of regulation would serve two functions: (1) the activating factor would not be introduced into the embryo until the time at which the eggshell was required, and (2) it would prevent CHS-1 from ectopically activating. A precociously assembled eggshell would be developmentally catastrophic because it would prevent fertilization.

In *Ascaris*, chitin synthase was found in two forms, an inactive form in unfertilized oocytes and an activated form converted by protease activity in fertilized embryos (Dubinsky et al., 1986). Identifying the protease that processes CHS-1 may help to identify other genes required for the process of eggshell formation and egg activation. The sperm may contain a protease necessary for chitin synthase activation, either by processing CHS-1 directly, by processing an inhibitor (which would lead to degradation), or by processing an activator (Merzendorfer, 2006). Evidence points to a serine or cysteine protease (Martinez-Rucobo et al., 2009). An analogous regulation is also found in *Manduca sexta* (insect) via a serine protease called CTLP1 (Broehan et al., 2007). The closest worm paralogs are *try-1*, *svh-1*, and *try-3.* Deletions exist for some of these genes but no reports of eggshell phenotypes have been made.

Are there additional paternal factors involved in eggshell formation? In order to identify such factors (i.e., the putative sperm protease), RNAi screens are not likely to be the most fruitful strategy since many sperm genes are refractory to RNAi-mediated knockdown. The analysis of the above-mentioned protease genes might prove fruitful, as well as the genetic and biochemical pursuit of proteins that act in the SPE-11 pathway. With the recent successes of CRISPR/Cas9-mediated genome editing in *C. elegans* (e.g., Arribere et al., 2014; Paix et al., 2014), one can directly create mutant alleles of candidates that might have paternal roles in eggshell formation.

Of the >100 genes already identified in RNAi screens as yielding the OID phenotype (Table 1, Section 12) (http://www.wormbase.org/; Sonnichsen et al., 2005), what hidden details exist in this collection of genes that would further clarify how the eggshell is synthesized and modified during early embryogenesis? Surely some of these genes will provide insights into the biosynthetic pathway for the permeability barrier. Another source of eggshell synthesis candidates may come from screens where genes involved in eggshell synthesis might be scored as maternal sterile or ugly brown Mels as well as OID (Green et al., 2011; A. Singson, personal communication). The depletion of CHS-1, for example, whether by RNAi or mutant analysis, results in such fragile embryos that the mothers may appear to be sterile. Thus, the examination of sterile mutants may uncover a few previously overlooked eggshell defective mutants.

Great strides have been made in the last 10 years to uncover the mechanism and composition of eggshell formation in *C. elegans*. The new ultrastructural information presented by Olson et al. (2012) should spur a reexamination of past data and a more focused investigation in the field going forward.

## 12. Table 1. Genes that when depleted cause the OID phenotype

Table 1 not only includes genes that have been scored as OID in RNAi screens, but also includes genes that have been scored as Passage through meiosis defective. We know that many of these genes are defective for a normal eggshell (e.g., the APC subunit genes). Though the causes for a defective eggshell are almost certainly indirect (metaphase I arrested zygotes fail to undergo CGE), we can still learn a great deal about the process of eggshell formation from the study of such genes. Near the bottom of this Table is a short list of genes implicated in eggshell function based on domains that they contain, such as chitin-binding domains, but that have not been identified as OID in RNAi assays. At the very bottom of this Table are five embryonic lethal genes that have not yet been molecularly identified but whose phenotypic characterization included OID.

## Figures and Tables

**Table 1:** Genes that when depleted cause the OID phenotype.

Coding Sequence/gene	Phenotype	RNAi citations	Alleles	Protein
nuclear pore				
*npp-20*	Embryo osmotic integrity defective	Sonnichsen et al., 2005	none	Nuclear Pore complex Protein
*ran-1*4	Embryo osmotic pressure sensitive	Piano et al., 2002	*tm5197*; lethal/sterile	Ran GTPase
**mitochondrial targeting**				
C47G2.3	Embryo osmotic integrity defective	Sonnichsen et al., 2005	*ok2557* deletion; larval lethal	
**F45G2.8**	Embryo osmotic integrity defective	Sonnichsen et al., 2005	none	
*sdha-1*	Passage through meiosis defective	Sonnichsen et al., 2005	2 deletions; lethal	Succinate DeHydrogenase complex subunit A
**ER targeting/folding**				
F38A1.8	Embryo osmotic integrity defective	Sonnichsen et al., 2005	none	signal recognition particle receptor subunit
R186.3	Embryo osmotic integrity defective	Sonnichsen et al., 2005	none	signal recognition particle receptor subunit
*phi-20*	Embryo osmotic integrity defective	Sonnichsen et al., 2005	none	
*cct-2*	Embryo osmotic integrity defective	Sonnichsen et al., 2005	*ok3438* deletion; lethal	Chaperonin Containing TCP-1
*cct-5*	Embryo osmotic integrity defective	Sonnichsen et al., 2005	none	Chaperonin
*hsp-4*	Embryo osmotic integrity defective	Sonnichsen et al., 2005	*gk514* deletion; viable	heat shock
*emc-2*	Embryo osmotic integrity defective	Sonnichsen et al., 2005	none	yeast EMC homolog
**cytoskeleton**				
*arp-11*	Embryo osmotic integrity defective	Sonnichsen et al., 2005	none	Actin-related protein
**extracellular**				
*sqt-1*	Embryo osmotic integrity defective	Sonnichsen et al., 2005	many; larval morphology mutants	collagen
*cbd-1*	Embryo osmotic integrity defective	Sonnichsen et al., 2005	*ok2913* deletion (in frame); viable	chitin binding domain protein
**glycosylation**				
*mig-22*	Embryo osmotic integrity defective	Sonnichsen et al., 2005	*tk69* deletion; lethal	abnormal cell MIGration
*sqv-4*	Embryo osmotic integrity defective	Sonnichsen et al., 2005	*n2827, n2840*; sterile/emb lethal	UDP-glucose 6-dehydrogenase
ZK686.3	Embryo osmotic integrity defective	Sonnichsen et al., 2005	none	
F09E5.2	Embryo osmotic integrity defective	Sonnichsen et al., 2005	none	Mannosyltransferase
*ribo-1*	Embryo osmotic integrity defective	Sonnichsen et al., 2005	none	Oligosaccharyltransferase
*ostb-1*	Embryo osmotic integrity defective	Sonnichsen et al., 2005	none	Oligosaccharyltransferase subunit
*ostd-1*	Embryo osmotic integrity defective	Sonnichsen et al., 2005	none	Oligosaccharyltransferase, Delta subunit
*dad-1*	Embryo osmotic integrity defective	Sonnichsen et al., 2005	none	inhibitor of cell death (Sugimoto *et al*., 1995)
*stt-3*	Embryo osmotic integrity defective	Sonnichsen et al., 2005	none	Oligosaccharyltransferase subunit
*ctps-1*	Embryo osmotic integrity defective	Sonnichsen et al., 2005	*gk38* deletion (in 5′ UTR); viable	CTP synthase
**hexosamine pathway**				
*gna-2*	Embryo osmotic integrity defective	Sonnichsen et al., 2005; Johnston et al., 2006	three deletions; lethal	glucosomine 6-phosphate N-acetyltransferase
F21D5.1	Embryo osmotic integrity defective	Sonnichsen et al., 2005	none	Phosphoacetylglucosamine mutase
F22B3.4	Embryo osmotic integrity defective	Piano et al., 2002; Sonnichsen et al., 2005	none	glucosamine-fructose 6-phosphate amino-transferase paralog
F07A11.2	Embryo osmotic integrity defective	Piano et al., 2002; Sonnichsen et al., 2005	none	glucosamine-fructose 6-phosphate amino-transferase paralog
C36A4.4	Embryo osmotic integrity defective	Sonnichsen et al., 2005	none	UDP-N-acetylglucosamine pyrophosphorylase
K06B9.2	Embryo osmotic integrity defective	Sonnichsen et al., 2005	none	hexosamine pathway
*chs-1*	maternal sterile/mel	many refs	*ok1120;* lethal	CHitin Synthase
**Chondroitin Proteolglycans**				
*cpg-1*	Embryo osmotic pressure sensitive	Johnston et al., 2006	*ok1914, ok1913*, 2 deletions	Chondroitin ProteoGlycan
*cpg-2*	Embryo osmotic pressure sensitive	Johnston et al., 2006	*ok2727* (viable?) not on OID list	Chondroitin ProteoGlycan
*sqv-5*	Embryo osmotic integrity defective	Olson et al., 2006	several alleles, 2 deletions; pleiotrophic phenotypes	SQuashed Vulva
**Fatty acids/lipid/sterol**				
*fasn-1*	Embryo osmotic integrity defective	Rappleye et al., 2003; Sonnichsen et al., 2005	none	fatty acid synthase
*cyp-31A1*	Embryo osmotic integrity defective	Piano et al., 2002; Sonnichsen et al., 2005	*gk154* deletion; viable	cytochrome P450
*cyp-31A2*	Embryo osmotic integrity defective	Piano et al., 2002; Sonnichsen et al., 2005	*tm2711* deletion; viable??	cytochrome P450 (Benenati *et al*., 2009)
*cyp-31A3*	Embryo osmotic pressure sensitive	Piano et al., 2002	*tm3224*; hypo-osmotic shock hyper-sensitive	cytochrome P450
*cyp-31A5*	Embryo osmotic integrity defective	Piano et al., 2002; Sonnichsen et al., 2005; Benenati et al., 2009	none	cytochrome P450
*emb-8*	Embryo osmotic integrity defective	Rappleye et al., 2003; Sonnichsen et al., 2005,	*hc69*; ts emb lethal	NADPH-cytochrome P450 reductase
*ptc-1*	Embryo osmotic integrity defective	Sonnichsen et al., 2005	none	PaTChed ortholog
*ptc-2*	Embryo osmotic integrity defective	Sonnichsen et al., 2005	none	PaTChed ortholog
*ptr-2*	Embryo osmotic integrity defective	Piano et al., 2002; Sonnichsen et al., 2005	*ok1338* deletion; lethal	PaTched-related
*hmgr-1*	Embryo osmotic integrity defective	Sonnichsen et al., 2005	*tm4368* deletion; lethal/sterile	HMG CoA reductase
*hmgs-1*	Embryo osmotic integrity defective	Sonnichsen et al., 2005	none	3-Hydroxy-3-Methyl-glutaryl CoA Synthase
*trcs-1*	Embryo osmotic integrity defective	Sonnichsen et al., 2005	none	putative arylacetamide deacetylase and microsomal lipase
*Ipin-1*	Embryo osmotic integrity defective	Sonnichsen et al., 2005	*ok2761* deletion; L1 arrest	LiPIN
*fdps-1*	Embryo osmotic integrity defective	Sonnichsen et al., 2005	none	Farnesyl diphosphate Synthetase
*dgtr-1*	Embryo osmotic integrity defective	Sonnichsen et al., 2005	none	Acyl-CoA diacylglycerol acyltransferase
*pod-2*	Embryo osmotic integrity defective	Rappleye et al., 2003	*ye60cs* allele; emb lethal	acetyl-CoA carboxylase
**trafficking**				
*arf-1.2*	Embryo osmotic integrity defective	Sonnichsen et al., 2005	*ok796* deletion; viable	ADP-ribosylation factor
*pod-1*	Embryo osmotic integrity defective	Sonnichsen et al., 2005	*gk516* deletion; sterile	Polarity and Osmotic sensitivity Defect
*rab-11.1*	Embryo osmotic integrity defective	Sonnichsen et al., 2005	5 *tm* deletions; sterile/lethal	RAB family
*sec-24.2*	Embryo osmotic integrity defective	Sonnichsen et al., 2005	none	yeast SEC homolog
*snap-1*	Embryo osmotic integrity defective	Sonnichsen et al., 2005	*tm2068* deletion allele; sterile/lethal	Soluble NSF attachment protein
*syx-5 syn-3*	Embryo osmotic integrity defective	Sonnichsen et al., 2005	none	SYntaXin, SNARE
*syx-4 syn-4*	Embryo osmotic integrity defective	Sonnichsen et al., 2005	two deletions; sterile	Syntaxin-related t-SNARE
*ykt-6*	Embryo osmotic integrity defective	Sonnichsen et al., 2005	*tm3575* deletion; larval lethal (Maekawa *et al.,* 2009)	SNARE protein
ZK1098.5	Embryo osmotic integrity defective	Sonnichsen et al., 2005	none	
*ggtb-1*	Embryo osmotic integrity defective	Sonnichsen et al., 2005	none	geranylgeranyltransferase
*rme-2*	Embryo osmotic integrity defective	Sonnichsen et al., 2005	*b1005* deletion, 2 point mutations; emb lethal	LDL receptor family
*cogc-4*	Embryo osmotic integrity defective	Sonnichsen et al., 2005	none	Conserved Oligomeric Golgi (COG) Component
*agef-1*	Embryo osmotic integrity defective	Sonnichsen et al., 2005	2 deletions; lethal/sterile – no strain	Arf-1 Guanine nucleotide Exchange Factor homolog
*sec-16*	Embryo osmotic integrity defective	Sonnichsen et al., 2005	none	yeast SEC homolog
**meiosis/mitosis**				
*cdk-1*	OID, Passage through meiosis defective	many refs	3 deletions; sterile	Cyclin-Dependent Kinase
*czw-1*	Embryo osmotic integrity defective	Sonnichsen et al., 2005	none	Caenorhabditis Zeste White 10
C27A2.3	Embryo osmotic integrity defective	Piano et al., 2002; Sonnichsen et al., 2005	*tm947* deletion; lethal/sterile	Interactor of FizzY; securin
*nud-1*	Embryo osmotic integrity defective	Piano et al., 2002; Sonnichsen et al., 2005	two deletions; one is sterile/lethal	Aspergillus NUclear Division related
*plk-1*	Embryo osmotic integrity defective	Sonnichsen et al., 2005	two deletion alleles and *or683ts*; lethal/sterile	POLO Kinase
*sep-1*	Embryo osmotic integrity defective	Sonnichsen et al., 2005	*ok1749* deletion; sterile	Separase
*wee-1.3*	Embryo osmotic integrity defective	Sonnichsen et al., 2005	few deletion alleles; larval lethal	Myt1 ortholog
*sds-22*	Embryo osmotic pressure sensitive	Piano et al., 2002	*tm5187*; lethal/sterile	conserved phosphatase regulator
*mei-1*	Passage through meiosis defective	Sonnichsen et al., 2005	missense and nonsense alleles; *ok2000* deletion; sterile	defective MEIosis; catalytic subunit of katanin
*mei-2*	Passage through meiosis defective	Sonnichsen et al., 2005	many alleles; Mel	defective MEIosis; targeting subunit of katanin
*cdc-25.1*	Passage through meiosis defective	Sonnichsen et al., 2005	*nr2036* deletion; sterile	CDC25 phosphatase ortholog
*fzy-1*	OID, Passage through meiosis defective	Sonnichsen et al., 2005, R ‘02	*ok312* deletion; sterile	Cdc20 ortholog; regulator of APC
*lsm-5cv*	Passage through meiosis defective	Sonnichsen et al., 2005	*ok3431* deletion; sterile	LSM Sm-like protein
*klp-10*	Passage through meiosis defective	Sonnichsen et al., 2005	2 deletions; viable	Kinesin-Like Protein
*klp-15*	Passage through meiosis defective	Sonnichsen et al., 2005	*ok1958* deletion; viable	Kinesin-Like Protein
*klp-18*	Passage through meiosis defective	Sonnichsen et al., 2005	3 deletions; sterile	Kinesin-Like Protein
*mesp-1*	Passage through meiosis defective	Sonnichsen et al., 2005	*tm2257* deletion; lethal/sterile – no strains	MEiotic Spindle
C02F5.12	Passage through meiosis defective	Sonnichsen et al., 2005	none	HIM-8 paralog
F52H2.7	Passage through meiosis defective	Sonnichsen et al., 2005	none	
F59E12.11	Passage through meiosis defective	Sonnichsen et al., 2005	*tm3828* deletion; lethal/sterile – no strain	
*imb-2*	Passage through meiosis defective	Sonnichsen et al., 2005	none	IMportin Beta family
*gld-1*	Passage through meiosis defective	Sonnichsen et al., 2005	*oz10* deletion; and many alleles	defective in Germ Line Development
*vbh-1*	Passage through meiosis defective	Sonnichsen et al., 2005	*ok567* deletion; lethal	Vasa- and Belle-like Helicase
*csn-4*	Passage through meiosis defective	Sonnichsen et al., 2005	4 deletions; lethal/sterile – no strains	COP-9 SigNalosome subunit
*csn-6*	Passage through meiosis defective	Sonnichsen et al., 2005	none	COP-9 SigNalosome subunit
Y57A10A.24	Passage through meiosis defective	Sonnichsen et al., 2005	2 deletions; viable	
Y57E12AL.6	Passage through meiosis defective	Sonnichsen et al., 2005	none	
**Ubiquitin/protein degradation**				
*mat-1*	Passage through meiosis defective	Sonnichsen et al., 2005	many ts alleles	Anaphase Promoting Complex
*mat-2*	Passage through meiosis defective	Sonnichsen et al., 2005	many ts alleles,; *gk824* deletion; lethal	Anaphase Promoting Complex
*mat-3*	OID, Passage through meiosis defective	Davis et al., 2002	many ts alleles	Anaphase Promoting Complex
*emb-1*	Passage through meiosis defective	Sonnichsen et al., 2005	ts allele, 3 deletion alleles; sterile/lethal	Anaphase Promoting Complex
*emb-27*	Passage through meiosis defective	Sonnichsen et al., 2005	many ts alleles	Anaphase Promoting Complex
*emb-30*	Embryo osmotic integrity defective	Sonnichsen et al., 2005	many ts alleles	Anaphase Promoting Complex
*apc-2*	Passage through meiosis defective	Sonnichsen et al., 2005	*ok1657* deletion; lethal	Anaphase Promoting Complex
*apc-11*	Passage through meiosis defective	Sonnichsen et al., 2005	*gk37* deletion; sterile	Anaphase Promoting Complex
*cdc-26*	Passage through meiosis defective	Sonnichsen et al., 2005	*ok1310* deletion; lethal	Anaphase Promoting Complex
*cdc-42*	Embryo osmotic integrity defective	Kay and Hunter, 2001	*gk388* deletion; sterile	Cell Division Cycle
*apc-17*	Embryo osmotic integrity defective	Sonnichsen et al., 2005	two deletion alleles; sterile	Anaphase Promoting Complex (Green *et al*., 2011 PMID 21529718)
*cyk-3*	Embryo osmotic integrity defective	Kaitna et al., 2002; Sonnichsen et al., 2005	none	CYtoKinesis defect
*atad-3*	Embryo osmotic pressure sensitive	Piano et al., 2002	*ok3093*; lethal	ATPase
*pas-1*	Passage through meiosis defective	Sonnichsen et al., 2005	*ok1531* deletion; lethal	Proteasome Alpha Subunit
*pas-2*	Passage through meiosis defective	Sonnichsen et al., 2005	*ok679* deletion; lethal	Proteasome Alpha Subunit
*pas-3*	Passage through meiosis defective	Sonnichsen et al., 2005	none	Proteasome Alpha Subunit
*pas-4*	Passage through meiosis defective	Sonnichsen et al., 2005	none	Proteasome Alpha Subunit
*pas-5*	Passage through meiosis defective	Sonnichsen et al., 2005	*ok1808* deletion; lethal	Proteasome Alpha Subunit
*pas-6*	Passage through meiosis defective	Sonnichsen et al., 2005	*tm1942* deletion; lethal/sterile – no strain	Proteasome Alpha Subunit
*pas-7*	Passage through meiosis defective	Sonnichsen et al., 2005	*ok3447* deletion; lethal	Proteasome Alpha Subunit
*pbs-1*	Passage through meiosis defective	Sonnichsen et al., 2005	none	Proteasome Beta Subunit
*pbs-2*	Passage through meiosis defective	Sonnichsen et al., 2005	none	Proteasome Beta Subunit
*pbs-3*	Passage through meiosis defective	Sonnichsen et al., 2005	none	Proteasome Beta Subunit
*pbs-4*	Passage through meiosis defective	Sonnichsen et al., 2005	*ok2856* deletion; lethal	Proteasome Beta Subunit
*pbs-5*	Passage through meiosis defective	Sonnichsen et al., 2005	*ok3318* deletion; lethal	Proteasome Beta Subunit
*pbs-6*	Passage through meiosis defective	Sonnichsen et al., 2005	2 deletions; early larval arrest	Proteasome Beta Subunit
*pbs-7*	Passage through meiosis defective	Sonnichsen et al., 2005	none	Proteasome Beta Subunit
*rpn-1*	Passage through meiosis defective	Sonnichsen et al., 2005	2 deletions; lethal	proteasome Regulatory Particle, Non-ATPase-like
*rpn-2*	Passage through meiosis defective	Sonnichsen et al., 2005	none	proteasome Regulatory Particle, Non-ATPase-like
*rpn-3*	Passage through meiosis defective	Sonnichsen et al., 2005	none	proteasome Regulatory Particle, Non-ATPase-like
*rpn-5*	Passage through meiosis defective	Sonnichsen et al., 2005	none	proteasome Regulatory Particle, Non-ATPase-like
*rpn-6*	Passage through meiosis defective	Sonnichsen et al., 2005	none	proteasome Regulatory Particle, Non-ATPase-like
*rpn-7*	Passage through meiosis defective	Sonnichsen et al., 2005	none	proteasome Regulatory Particle, Non-ATPase-like
*rpn-8*	Passage through meiosis defective	Sonnichsen et al., 2005	none	proteasome Regulatory Particle, Non-ATPase-like
*rpn-11*	Passage through meiosis defective	Sonnichsen et al., 2005	none	proteasome Regulatory Particle, Non-ATPase-like
*rpt-1*	Passage through meiosis defective	Sonnichsen et al., 2005	none	proteasome Regulatory Particle, ATPase-like
*rpt-2*	Passage through meiosis defective	Sonnichsen et al., 2005	none	proteasome Regulatory Particle, ATPase-like
*rpt-3*	Passage through meiosis defective	Sonnichsen et al., 2005	none	proteasome Regulatory Particle, ATPase-like
*rpt-4*	Passage through meiosis defective	Sonnichsen et al., 2005	2 deletions; lethal/sterile – no strains	proteasome Regulatory Particle, ATPase-like
*rpt-5*	Passage through meiosis defective	Sonnichsen et al., 2005	none	proteasome Regulatory Particle, ATPase-like
*uba-1*	Passage through meiosis defective	Sonnichsen et al., 2005	*ok1374* deletion; lethal	UBA (human ubiquitin) related
*ubl-1*	Embryo osmotic integrity defective	Sonnichsen et al., 2005	none	UBiquitin-Like
*ubq-1*	Passage through meiosis defective	Sonnichsen et al., 2005	none	UBiQuitin
*ubq-2*	Passage through meiosis defective	Sonnichsen et al., 2005	*ok2028* deletion; lethal	UBiQuitin
**Signaling**	Embryo osmotic integrity defective	Sonnichsen et al., 2005	*tm4868* deletion and many missense mutations	MAP kinase kinase
*mek-2*	Embryo osmotic integrity defective	Sonnichsen et al., 2005	*dx84* deletion; sterile/lethal	raf ortholog
*lin-45*	Embryo osmotic integrity defective	Sonnichsen et al., 2005	2 deletions; lethal	human TGF related
*tfg-1*				
**transcription/translation**				
*cgh-1*	Embryo osmotic integrity defective	Sonnichsen et al., 2005	*ok492* deletion; sterile	Conserved Germline RNA Helicase
*let-711*	Embryo osmotic integrity defective	Sonnichsen et al., 2005	none	NOT1 ortholog
*snr-1*	Embryo osmotic integrity defective	Sonnichsen et al., 2005	none	Small Nuclear Ribonucleoprotein
*snr-7*	Embryo osmotic integrity defective	Sonnichsen et al., 2005	none	Small Nuclear Ribonucleoprotein
*atx-2*	Embryo osmotic integrity defective	Sonnichsen et al., 2005	3 deletion strains; lethal/sterile – no strain	human ataxin-related
*gld-2*	Embryo osmotic integrity defective	Sonnichsen et al., 2005	none	defective in Germ Line Development
*puf-3*	Embryo osmotic integrity defective	Sonnichsen et al., 2005	none	PUF (Pumilio/FBF) domain-containing
*pab-1*	Embryo osmotic integrity defective	Sonnichsen et al., 2005	*ok1656* deletion; lethal	PolyA Binding protein
*ccf-1*	Embryo osmotic integrity defective	Sonnichsen et al., 2005	*gk40* deletion; lethal/sterile	yeast CCR4 associated Factor
*fars-1*	Embryo osmotic integrity defective	Sonnichsen et al., 2005	none	Phenylalanyl Amino-acyl tRNA Synthetase
*pars-1*	Embryo osmotic integrity defective	Sonnichsen et al., 2005	none	Prolyl Amino-acyl tRNA Synthetase
*rps-16*	Embryo osmotic integrity defective	Sonnichsen et al., 2005	none	small ribosomal subunit S16 protein
*rla-0*	Embryo osmotic integrity defective	Sonnichsen et al., 2005	*tm2743* deletion; lethal/sterile	Ribosomal protein, Large subunit
*rpl-24.2*	Embryo osmotic integrity defective	Sonnichsen et al., 2005	none	Ribosomal protein, Large subunit
*rpl-1*	Embryo osmotic integrity defective	Sonnichsen et al., 2005	none	Ribosomal Protein, Large subunit
*rpl-30*	Embryo osmotic integrity defective	Sonnichsen et al., 2005	*ok3566*; larval arrest	large ribosomal subunit L30
*eif-3.C*	Embryo osmotic integrity defective	Sonnichsen et al., 2005	none	translation initiation factor 3 subunit C
*eif-3.I*	Embryo osmotic integrity defective	Sonnichsen et al., 2005	none	Eukaryotic Initiation Factor
*eef-2*	Embryo osmotic integrity defective	Sonnichsen et al., 2005	*ok1774* deletion; lethal	translation elongation factor 2
*erfa-1*	Embryo osmotic integrity defective	Sonnichsen et al., 2005	none	Peptide chain release factor 1
**ATP**				
*atp-3*	Embryo osmotic integrity defective	Sonnichsen et al., 2005	none	ATP synthase subunit
**ATPase**				
*vha-4*	Embryo osmotic integrity defective	Sonnichsen et al., 2005	none	Vacuolar H ATPase
*vha-10*	Embryo osmotic integrity defective	Sonnichsen et al., 2005	none	Vacuolar H ATPase
*vha-11*	Embryo osmotic integrity defective	Sonnichsen et al., 2005	none	Vacuolar H ATPase
*vha-12*	Embryo osmotic integrity defective	Sonnichsen et al., 2005	*ok821* deletion allele; viable	Vacuolar H ATPase
*vha-14*	Embryo osmotic integrity defective	Sonnichsen et al., 2005	none	Vacuolar H ATPase
*vha-17*	Embryo osmotic integrity defective	Sonnichsen et al., 2005	none	Vacuolar H ATPase
*vha-19*	Embryo osmotic integrity defective	Sonnichsen et al., 2005	*tm2225* deletion; viable	Vacuolar H ATPase
**uncharacterized**				
*dhc-3*	Embryo osmotic integrity defective	Sonnichsen et al., 2005	none	microtubule associated protein (Wolke et al., 2007)
*egg-6*	Embryo osmotic integrity defective	Sonnichsen et al., 2005	*ok1506* deletion; lethal	extracellular leucine-rich repeat protein
*ifet-1*	Embryo osmotic integrity defective	Sonnichsen et al., 2005	2 deletions; sterile/lethal	Prion-like-(Q/N-rich)-domain-bearing protein
*perm-1*	Embryo osmotic integrity defective	Sonnichsen et al., 2005	none	PERMeable eggshell
*perm-2*	Embryo osmotic integrity defective	Sonnichsen et al., 2005	none	PERMeable eggshell
*perm-3*	Embryo osmotic integrity defective	Sonnichsen et al., 2005	*ok2654* deletion; lethal	PERMeable eggshell
*perm-5*	Embryo osmotic integrity defective	Sonnichsen et al., 2005	*tm4637* deletion; lethal/sterile	PERMeable eggshell
*phi-62*	Embryo osmotic integrity defective	Sonnichsen et al., 2005	none	
*rmd-1*	Embryo osmotic integrity defective	Sonnichsen et al., 2005	*tm1457* deletion; sterile/lethal	Regulator of Microtubule Dynamics
*smgl-1*	Embryo osmotic integrity defective	Sonnichsen et al., 2005	*ok2423* deletion; larval lethal	SMG-associated
*vps-4*	Embryo osmotic integrity defective	Sonnichsen et al., 2005	none	related to yeast Vacuolar Protein Sorting factor
Y51A2A.4	Embryo osmotic integrity defective	Sonnichsen et al., 2005	none	
T01C3.11	Embryo osmotic integrity defective	Sonnichsen et al., 2005	none	
C31H1.8	Embryo osmotic pressure sensitive	Piano et al., 2002	none	
H02I12.5	Embryo osmotic integrity defective	Sonnichsen et al., 2005	*tm3158*; no phenotype reported	
E02H9.3	Embryo osmotic integrity defective	Sonnichsen et al., 2005	none	
T05G5.9	Embryo osmotic integrity defective	Sonnichsen et al., 2005	*tm1457*; lethal/sterile	
F57B10.14	Embryo osmotic integrity defective	Sonnichsen et al., 2005	none	
K10F12.6	Embryo osmotic integrity defective	Sonnichsen et al., 2005	none	
**Implicated in eggshell function from the literature**				
*egg-3*	maternal sterile	many refs	2 deletions; sterile	EGG sterile (unfertilizable)
*gale-1*	no RNAi phenotypes reported	J Johnston and Dennis, 2012	*tm3267* deletion; lethal/sterile – no strains	UDP-GALactose 4-Epimerase
*gpi-1*	embryonic lethal	Sonnichsen et al., 2005	*ok3599* deletion; lethal	Glucose-6-Phosphate Isomerase
*pqn-74*	no embryo phenotype by RNAi	Johnston et al., 2006	none; no phenotype	chitin-binding domains
*inx-9*	permeable eggshell	Carvalho et al., 2011		INneXin gap junction protein
C39D10.7	no embryo phenotype by RNAi	Johnston et al., 2006	*ok2758* deletion; viable; transcripts enriched in herma-phrodite	chitin-binding domains
F23F12.8	no embryo phenotype by RNAi	Johnston et al., 2006	none; expressed in pharynx	chitin-binding domains
K04H4.2	larval arrest	many; Johnston et al., 2006	*tm4705* deletion; lethal/sterile – no strains	chitin-binding domains
M03E7.4	no RNAi phenotypes reported	Johnston et al., 2006	none; no phenotype	chitin-binding domains
R02F2.4	no RNAi phenotypes reported	Johnston et al., 2006	none; no phenotype; message is expressed in the oocyte	chitin-binding domains; CPG-2-related
T10E10.4	no RNAi phenotypes reported	Johnston et al., 2006	none; similar to K04H4.2 but no RNAi or expression data	chitin-binding domains
W03F11.1	maternal sterile by RNAi	Maeda et al., 2001; Johnston et al., 2006	none	chitin-binding domains
**Uncloned genetic loci**	OID (mutant)	Isnenghi et al., 1983	ts allele	abnormal EMBroygenesis
*emb-11*	OID (mutant)	Isnenghi et al., 1983	ts allele	abnormal EMBroygenesis
*emb-12*	OID (mutant)	Isnenghi et al., 1983	ts allele	abnormal EMBroygenesis
*emb-14*	OID (mutant)	Isnenghi et al., 1983	ts allele	abnormal EMBroygenesis
*emb-19*	OID (mutant)	Isnenghi et al., 1983	ts allele	abnormal EMBroygenesis
*emb-20*				

Table 1 not only includes genes that have been scored as OID in RNAi screens, but also includes genes that have been scored as Passage through meiosis defective. We know that many of these genes are defective for a normal eggshell (i.e., the APC subunit genes). Though the causes for a defective eggshell are almost certainly indirect (metaphase I arrested embryos fail to undergo CGE), we can still learn a great deal about the process of eggshell formation from the study of such genes. Near the bottom of this Table is a short list of genes implicated in eggshell function based on domains that they contain, such as chitin-binding domains, but that have not been identified as OID in RNAi assays. At the very bottom of this Table are five embryonic lethal genes that have not yet been molecularly identified but whose phenotypic characterization included OID.
